# Bidirectional Relationship and Shared Mechanisms Between Sarcopenia and Osteoporosis: An Observational Study Integrating Genomic, Proteomic, and Metabolomic Data

**DOI:** 10.1111/acel.70617

**Published:** 2026-06-30

**Authors:** Siqi Xu, Simin Wen, Xizeng Zong, Jianwei Zhu, Qiuling Du, Yu Li, Kunyan Wang, Peihua Cao, Changhai Ding, Yan Zhang, Guangfeng Ruan

**Affiliations:** ^1^ Department of Orthopedics, School of Medicine The Second Affiliated Hospital of South China University of Technology (Guangzhou First People's Hospital) Guangzhou China; ^2^ Clinical Research Centre Guangzhou First People's Hospital, Guangzhou Medical University Guangzhou China; ^3^ School of Medicine South China University of Technology Guangzhou China; ^4^ Clinical Research Centre Zhujiang Hospital, Southern Medical University Guangzhou China; ^5^ Menzies Institute for Medical Research University of Tasmania Hobart Australia; ^6^ Clinical Research Centre Beijing Tsinghua Changgung Hospital, Tsinghua Medicine, Tsinghua University Beijing China

**Keywords:** bidirectional association, multi‐omics analysis, osteoporosis, sarcopenia

## Abstract

With global population aging, sarcopenia and osteoporosis have become critical public health challenges. Muscles and bones are closely interconnected anatomically and functionally, but the biological mechanisms connecting sarcopenia and osteoporosis have not yet been fully elucidated. This study systematically investigated the bidirectional relationship and shared mechanisms between sarcopenia and osteoporosis through multi‐omics analysis integrating genomic, proteomic, and metabolomic data from the UK Biobank. Our findings indicated that the two conditions may act as reciprocal risk factors. Higher lean mass index (hazard ratio [HR] = 0.830, 95% confidence interval [CI]: 0.788–0.874), greater hand grip strength (HR = 0.544, 95% CI: 0.531–0.558) and faster usual walking pace (HR = 0.735, 95% CI: 0.723–0.746) were significantly associated with lower osteoporosis incidence, with multiple plasma proteins and metabolites identified as mediators in these associations. Conversely, higher bone mineral density of left heel (HR = 0.613, 95% CI: 0.394–0.946) and right heel (HR = 0.591, 95% CI: 0.380–0.912) were associated with reduced sarcopenia risk. We also observed a positive genetic correlation (*r* = 0.25, *p* = 1.6 × 10^−5^) between sarcopenia and osteoporosis, as well as substantial overlaps in risk‐associated genes and circulating biomarkers between the two conditions with consistent effect directions. Furthermore, modifiable factors including smoking, sleep duration, and physical activity exhibited similar effect patterns between sarcopenia and osteoporosis, with more than 30% overlap in mediating proteins and metabolites. These discoveries highlight common pathophysiological pathways potentially underlying both conditions and are expected to advance the understanding of muscle‐bone interdependence.

## Graphical abstract

This study investigated the bidirectional relationship and shared mechanisms between sarcopenia and osteoporosis using data from the UK Biobank. Our findings indicated that the two conditions may act as reciprocal risk factors, with multiple potential biomarkers mediating the muscle‐bone relationship and suggested possible shared signals between the two diseases.

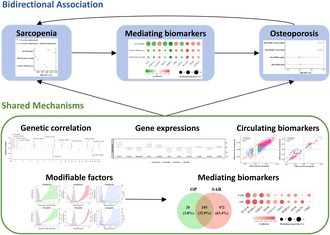

## 1 Introduction

With the accelerating global aging population, degenerative musculoskeletal disorders have become a critical public health concern (Nguyen et al. 2025). Sarcopenia, a progressive syndrome characterized by the loss of skeletal muscle mass, strength, and function, significantly increases the risk of falls, fractures, and disability in older adults, leading to severe clinical consequences (Chung et al. 2025). Epidemiological studies indicate that the prevalence of sarcopenia in older adults ranges from 10% to 27%, depending on the diagnostic criteria applied (Petermann‐Rocha et al. 2022). According to the World Health Organization, the population aged 60 and above is predicted to reach 2 billion by 2050, and with conservative estimates, over 200 million individuals worldwide will be affected by sarcopenia (Cruz‐Jentoft et al. 2010).

Concurrently, osteoporosis—a systemic skeletal disorder marked by reduced bone mass, deteriorated microarchitecture, and increased fragility—often results in fractures, chronic pain, and mobility impairment, profoundly impacting the quality of life in middle‐aged and elderly populations (Rachner et al. 2011). The estimated global prevalence of osteoporosis in older adults is up to 21.7% (Salari et al. 2021). In European countries, the economic burden of osteoporotic fractures was estimated to reach €37 billion in 2010 and projected to increase by 25% in 2025 (Hernlund et al. 2013).

Muscles and bones share a common embryological origin from mesodermal and ectodermal mesenchymal stem cells and maintain close anatomical and functional interactions (DiGirolamo et al. 2013; Gielen et al. 2023). Recent studies have revealed a bidirectional relationship between muscle and bone health: mechanical loading from muscles stimulates bone formation and maintains bone mineral density (BMD) (Novotny et al. 2015), while reduced BMD has been identified as a risk factor for sarcopenia (Cheng and Wang 2023). The concurrent occurrence of sarcopenia and osteoporosis leads to a condition known as “osteosarcopenia,” characterized by simultaneous declines in muscle mass and function, as well as bone density and strength (Teng et al. 2021). Patients with osteosarcopenia face an elevated fracture risk compared to those with either condition alone (Chen, Xu, et al. 2024; Yu et al. 2014). However, the underlying mechanisms linking sarcopenia and osteoporosis remain incompletely understood. Thus, elucidating the reciprocal relationship and shared pathological pathways between sarcopenia and osteoporosis holds significant scientific and clinical value.

In this study, we performed a comprehensive multilevel analysis of the sarcopenia‐osteoporosis relationship using UK Biobank data. By integrating multi‐omics approaches including genomic, proteomic, and metabolomic profiling, we sought to (1) systematically evaluate bidirectional associations between sarcopenia and osteoporosis; (2) identify key plasma protein and metabolite mediators of their pathophysiological interplay; (3) characterize shared molecular mechanisms, including genetic architecture, protein and metabolite signatures, and transcriptional regulation underlying both conditions; and (4) investigate common lifestyle factors influencing both diseases while delineating biological pathways through which modifiable factors affect disease risks, thereby advancing the understanding of muscle‐bone interdependence and revealing potential targets for combined therapeutic strategies against sarcopenia and osteoporosis.

## 2 Results

### 2.1 Sarcopenia and Osteoporosis May Be Reciprocal Risk Factors

**FIGURE 1:**
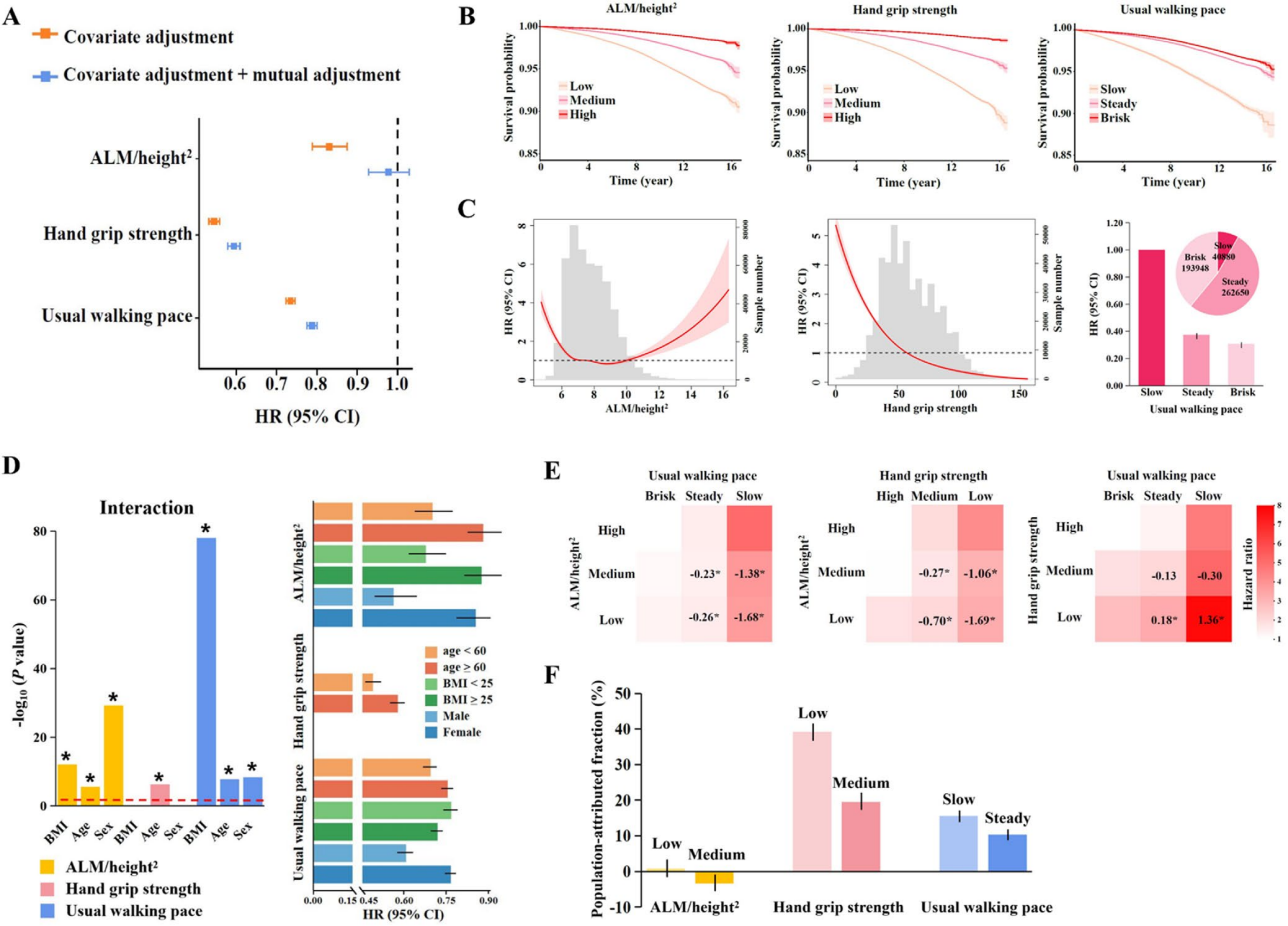
Longitudinal associations between sarcopenia traits and osteoporosis risk. (A) Hazard ratios (HRs) and 95% confidence intervals (CIs) for the associations of each sarcopenia trait with osteoporosis risk. Covariates adjustment: adjusting for sex, age, ethnicity, and BMI; Mutual adjustment: adjusting for the other two sarcopenia traits. (B) Survival curves for osteoporosis onset stratified by sarcopenia trait levels. (C) Left and Middle: Exposure–response curves depict HRs and 95% CIs for osteoporosis risk against ALM/height^2^ and hand grip strength under restricted cubic spline regressions. Histograms display sample distributions of each trait. Right: Bar plot presents HRs and 95% CIs for osteoporosis risk by categories of usual walking pace. Pie chart shows sample distribution of usual walking pace. (D) Left: *p* values for the effects of multiplicative interactions between sarcopenia traits and covariates on osteoporosis risk (red dashed line: *p* = 0.05). Right: HRs and 95% CIs for associations between sarcopenia traits and osteoporosis risk stratified by age, BMI, and sex. (E) HRs for osteoporosis risk across pairwise combinations of sarcopenia; numeric values represent the relative excess risk due to interaction (reference: lowest levels of both traits). (F) Population attributable fractions of sarcopenia traits for osteoporosis risk (reference: highest trait level). ALM, appendicular lean mass; BMI, body mass index. *indicates significant interaction.

To study the influence of sarcopenia on osteoporosis, we examined the associations of three sarcopenia traits, including appendicular lean mass (ALM) normalized to height squared (ALM/height^2^), hand grip strength, and usual walking pace, with osteoporosis incidence. Longitudinal analyses demonstrated that higher ALM/height^2^ (HR = 0.830, 95% CI: 0.788–0.874), hand grip strength (HR = 0.544, 95% CI: 0.531–0.558), and faster usual walking pace (HR = 0.735, 95% CI: 0.723–0.746) were significantly associated with a reduced risk of osteoporosis. After mutual adjustment for these sarcopenia traits, both hand grip strength (HR = 0.593, 95% CI: 0.578–0.608) and usual walking pace (HR = 0.788, 95% CI: 0.776–0.800) remained independent protective factors of osteoporosis (Figure 1A; Table S1). Higher levels of the three sarcopenia traits also exhibited increased osteoporosis‐free survival probabilities, respectively (Figure 1B). We also explored the dose–response relationships between sarcopenia traits and osteoporosis risk. Higher hand grip strength and faster walking pace were monotonically associated with reduced osteoporosis risk, whereas ALM/height^2^ exhibited a U‐shaped relationship with osteoporosis risk (Figure 1C).

We observed significant multiplicative interactions of covariates (sex, age, and body mass index [BMI]) with ALM/height^2^ and usual walking pace, as well as age with hand grip strength (Figure 1D; Table S2). Stratified analyses revealed that all the three sarcopenia traits showed stronger associations with osteoporosis risk in younger adults. ALM/height^2^ and usual walking pace had greater impact on osteoporosis risk in males than females. For individuals with lower BMI, ALM/height^2^ exhibited larger effect but usual walking pace had weaker effect on osteoporosis risk. Notably, despite the presence of multiplicative interactions, favorable sarcopenia traits were consistently associated with a significantly lower risk of osteoporosis in these subgroups (Figure 1D; Table S3). Additionally, using pairwise combinations of high ALM/height^2^, brisk walking pace and high hand grip strength as the reference, additive interaction analyses revealed antagonistic interactions for ALM/height^2^ with walking pace and hand grip strength, attenuating their combined adverse effects on osteoporosis. In contrast, a synergistic interaction was observed for low hand grip strength with steady and slow walking pace, amplifying their combined detrimental impact on osteoporosis (Figure 1E; Table S4). Population attributable fraction (PAF) analysis showed that lower hand grip strength and slower walking pace had higher PAF for osteoporosis, while medium ALM/height^2^ exhibited the lowest PAF for osteoporosis (Figure 1F; Table S5). We also observed significant longitudinal associations of sarcopenia traits (except ALM/height^2^) at the imaging visit, as well as sarcopenia diagnosis at baseline, with osteoporosis risk (Table S6). Cross‐sectional analyses further supported the baseline associations of sarcopenia traits and diagnosis with osteoporosis prevalence (Table S7). Additionally, Mendelian randomization confirmed causal effects of most sarcopenia traits (except usual walking pace) on BMD of lumbar spine (Table S8).

To evaluate the influence of osteoporosis on sarcopenia, we used heel BMD (i.e., left and right heel BMD and BMD *T*‐scores) as osteoporosis traits and identified sarcopenia cases by “probable sarcopenia” (see Section 5 for the definition) in our primary analysis unless otherwise specified. Longitudinal analysis revealed that higher osteoporosis traits were associated with reduced sarcopenia risk (Table S9). Restricted cubic spline regressions further demonstrated monotonic inverse relationships between osteoporosis traits and sarcopenia risk (Figure S1). We did not observe significant multiplicative interactions between osteoporosis traits and the covariates (sex, age, BMI) (Table S10), or additive interaction between left and right heel BMD categories (classified by BMD *T*‐scores as normal, osteopenia, or osteoporosis) for sarcopenia risk (Table S11). PAF analysis demonstrated that osteopenia and osteoporosis in heels had limited population‐level impact on sarcopenia (Table S12). Although longitudinal association between osteoporosis diagnosis and sarcopenia risk was not observed, cross‐sectional analyses supported the associations of osteoporosis traits and diagnosis with sarcopenia prevalence (Tables S13 and S14). Moreover, we conducted complementary analyses using “confirmed sarcopenia” and femoral neck/lumbar spine BMD (see Section 5 for details). The results showed that osteoporosis diagnosis was significantly associated with confirmed sarcopenia in longitudinal analysis (Table S15). In cross‐sectional analysis at the imaging visit, both femoral neck BMD and lumbar spine BMD also showed significant negative associations with probable sarcopenia and confirmed sarcopenia (Table S15). Mendelian randomization further revealed causal effects of lumbar spine BMD on most sarcopenia traits (except usual walking pace) (Table S16).

### 2.2 Proteins and Metabolites May Mediate the Effect of Muscle on Osteoporosis

**FIGURE 2:**
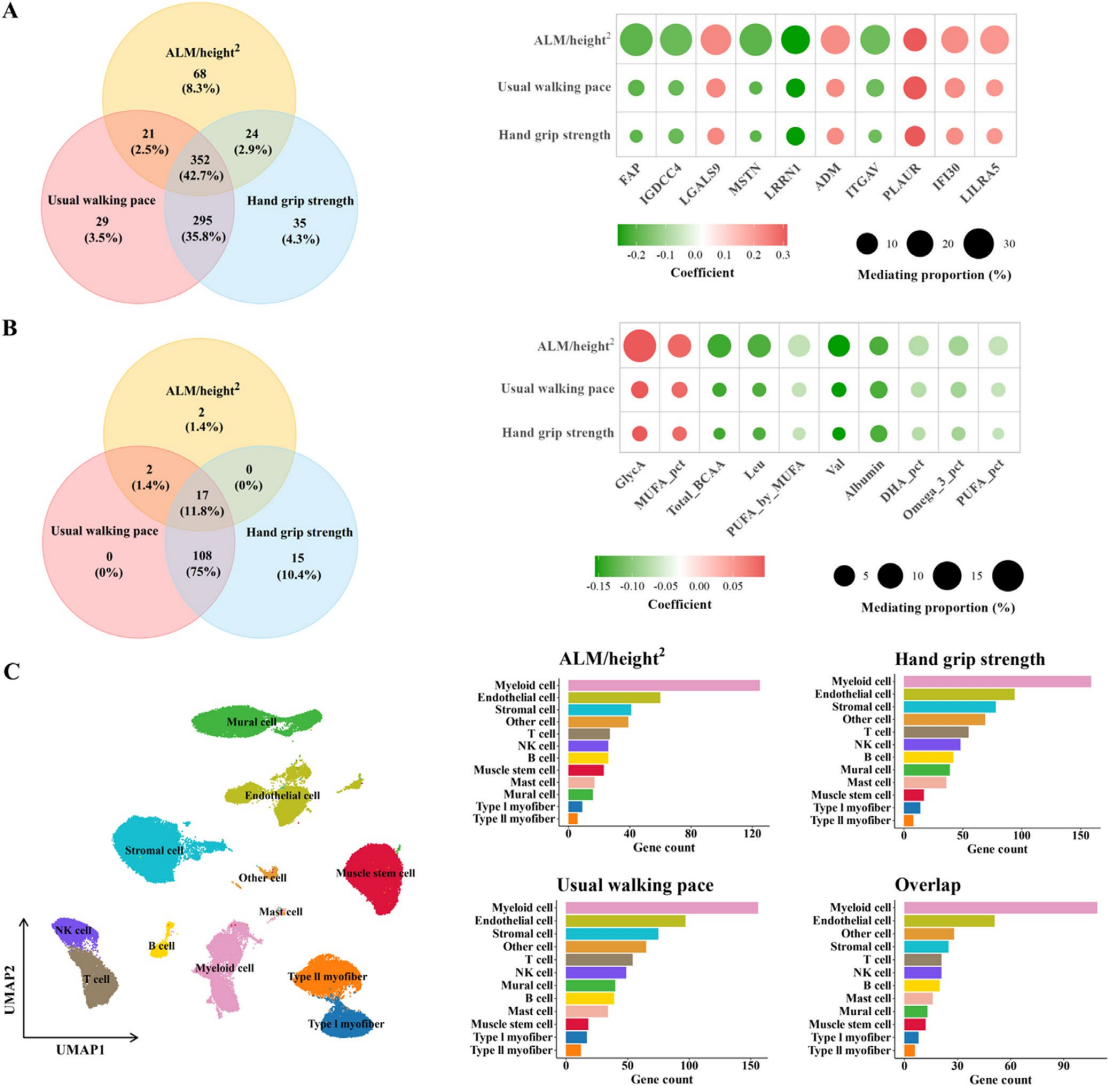
Analyses for mediating proteins and metabolites in the associations between sarcopenia traits and osteoporosis risk. (A, B) Left: Venn diagrams showing the counts of significant mediating (A) proteins and (B) metabolites for each sarcopenia trait. Right: Bubble plots showing top 10 significant mediators with the largest sum of mediating proportions across sarcopenia traits, with colors indicating effect sizes of standardized protein/metabolite levels on osteoporosis risk. (C) Left: Visualization of cell clusters and annotated cell types using scRNA‐seq data from skeletal muscle based on uniform manifold approximation and projection (UMAP). Right: Expression profiles of trait‐specific or overlapping mediating proteins for sarcopenia traits across the enriched cell types. ALM, appendicular lean mass.

We performed mediation analyses to identify plasma proteins and metabolites that mediate the effects of sarcopenia traits on osteoporosis risk. Protein analyses revealed 465 mediators for ALM/height^2^, 706 for hand grip strength, and 697 for usual walking pace, with 352 shared proteins showing mediation effects for three sarcopenia traits (Figure 2A; Tables S17–S19). Metabolite analyses identified 21 mediators for ALM/height^2^, 140 for hand grip strength, and 127 for usual walking pace, including 17 metabolic mediators that overlapped among three sarcopenia traits (Figure 2B; Tables S20–S22).

Muscles may influence skeleton through the secretion of proteins. Therefore, we investigated cellular origins of the proteins mediating the associations between sarcopenia traits and osteoporosis risk using single‐cell RNA sequencing (scRNA‐seq) data comprising 12 distinct cell types from skeletal muscle (Figure 2C; Figure S2 and Table S23). Our analyses demonstrate that myeloid cells, endothelial cells, and stromal cells in skeletal muscle showed predominant enrichment of the mediating proteins for three sarcopenia traits, with myeloid cells exhibiting the strongest enrichment for the mediating proteins shared among sarcopenia traits (Figure 2C; Tables S24–S27).

However, due to insignificant longitudinal associations between osteoporosis traits and sarcopenia risk in subpopulations with available proteomic and metabolomic data (Table S9), we did not perform mediation analyses for these relationships.

### 2.3 Shared Genetic Architecture of Sarcopenia and Osteoporosis

**FIGURE 3:**
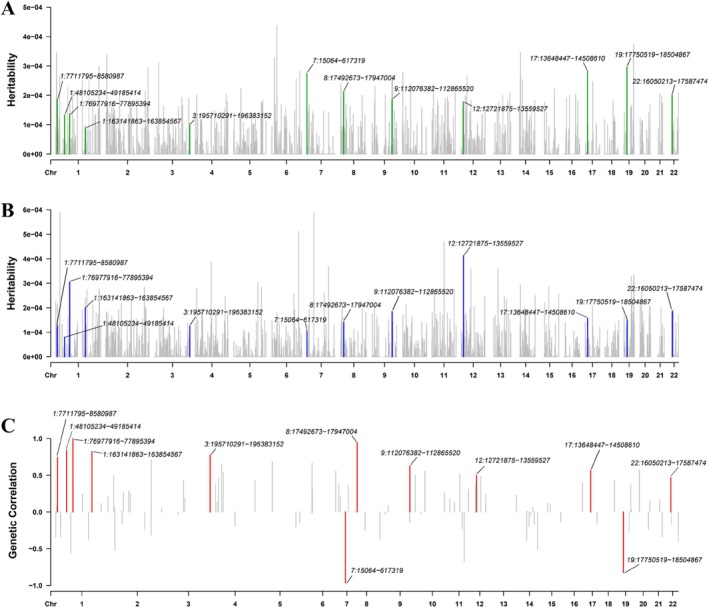
Manhattan plots of local heritability and genetic correlation for sarcopenia and osteoporosis across genomic loci. (A) Local heritability estimates of sarcopenia. (B) Local heritability estimates of osteoporosis. (C) Local genetic correlation estimates between sarcopenia and osteoporosis in genomic loci with significant heritability of both sarcopenia and osteoporosis. Colored bars (green, blue, and red) represent genomic loci that showed significant local genetic correlations.

To investigate whether sarcopenia and osteoporosis share common genetic architecture, we perform linkage disequilibrium score (LDSC) regression. The analysis revealed significant heritability of sarcopenia (*h* ^2^ = 0.017, *p* = 1.9 × 10^−32^) and osteoporosis (*h* ^2^ = 0.019, *p* = 1.8 × 10^−22^), and demonstrated a significant positive genetic correlation between sarcopenia and osteoporosis risks across the genome (*r* = 0.25, *p* = 1.6 × 10^−5^). Additionally, local genetic correlation analysis by the Local Analysis of [co]Variant Association (LAVA) method identified 102 genomic regions with significant heritability for both sarcopenia and osteoporosis. Among these, 12 genomic regions showed significant genetic correlations between the two diseases, with 10 genomic regions exhibiting positive correlations (Figure 3; Figure S3 and Tables S28 and S29).

We further examined the association of polygenic risk scores (PRS) of osteoporosis with sarcopenia risk, as well as the association of sarcopenia PRS with osteoporosis risk. The analyses showed that sarcopenia PRS was associated with osteoporosis risk in both cross‐sectional analysis (OR = 1.074, *p* = 3.7 × 10^−17^) and longitudinal analysis (HR = 1.051, *p* = 2.2 × 10^−58^). Similarly, osteoporosis PRS exhibited significant associations with both the prevalence (OR = 1.037, *p* = 7.4 × 10^−56^) and incidence (OR = 1.02, *p* = 2.4 × 10^−4^) of sarcopenia (Table S30).

### 2.4 Overlap of Risk Proteins and Metabolites in Sarcopenia and Osteoporosis

**FIGURE 4:**
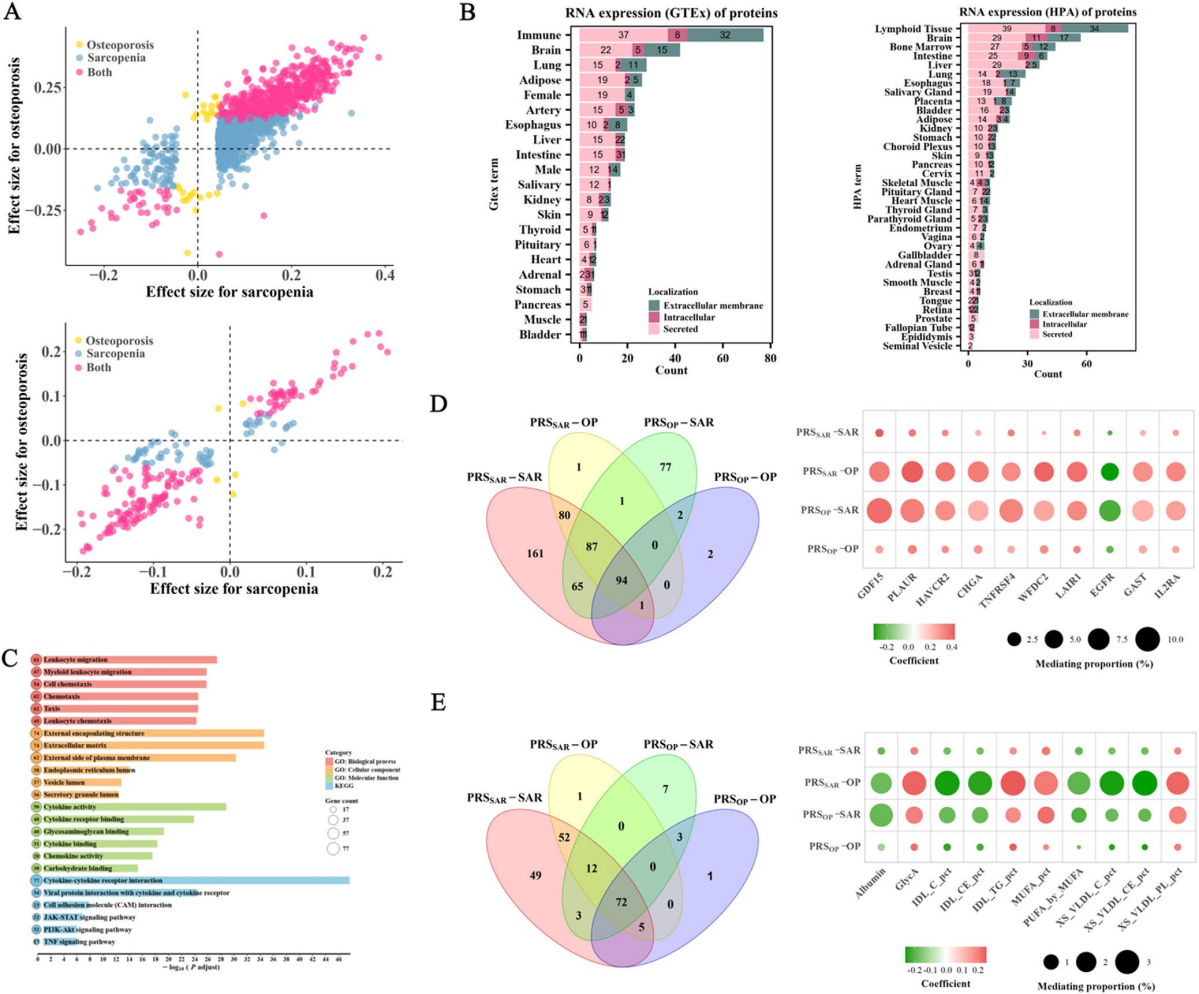
Shared proteomic and metabolomic signatures of sarcopenia and osteoporosis. (A) Scatter plots for effect sizes of standardized protein (top) and metabolite (bottom) levels on osteoporosis risk (*y*‐axis) and sarcopenia risk (*x*‐axis). Yellow: Osteoporosis‐specific associations; Blue: Sarcopenia‐specific associations; Red: Associations with both diseases (FDR < 0.05). (B) Tissue mapping (based on GTEx and HPA databases) and subcellular localization for proteins linked to both diseases in consistent effect directions. (C) Significantly enriched GO and KEGG pathways for proteins associated with both diseases in consistent effect directions (top five of three GO subcategories and KEGG). (D, E) Significant mediating (D) proteins and (E) metabolites in the PRS‐disease associations. Left: Venn diagrams for counts of significant mediators. Right: Top 10 significant mediators shared across four PRS‐disease associations with the largest sum of mediating proportions, with colors indicating effect sizes of standardized mediators on diseases' risks. GO, Gene Ontology; GTEx, Genotype‐Tissue Expression project; HPA, Human Protein Atlas database; KEGG, Kyoto Encyclopedia of Genes and Genomes; OP, osteoporosis; PRS_OP_, polygenic risk score of osteoporosis; PRS_SAR_, polygenic risk score of sarcopenia; SAR, sarcopenia.

To identify circulating biomarkers potentially influencing both osteoporosis and sarcopenia, we examined the associations of plasma proteins and metabolites with these two diseases. Our analysis revealed 226 metabolic biomarkers linked to sarcopenia prevalence and 169 to osteoporosis prevalence. Among these, 164 (71%) metabolic biomarkers were associated with both sarcopenia and osteoporosis, all exhibiting consistent effect directions to both diseases (Figure 4A; Table S31). We also identified 1576 sarcopenia‐associated and 530 osteoporosis‐associated proteins, with 502 (31.3%) associated with both sarcopenia and osteoporosis and 500 showing concordant effect directions to both diseases (Figure 4A; Table S32).

Bioinformatics analyses revealed that most overlapping proteins between the two diseases were secretory proteins, with their RNA expression predominantly enriched in immune and lymphoid tissues (Figure 4B; Tables S33 and S34), suggesting that immune‐related protein may underlie the shared pathophysiology of sarcopenia and osteoporosis. Multiple immune and inflammatory pathways were also significantly enriched for these overlapping proteins (Figure 4C; Tables S35 and S36). Protein–protein interaction (PPI) networks showed significant connectivity (*p* < 1 × 10^−16^), with immune‐regulatory hub proteins (e.g., IL6, CXCL8, and TNF) emerging as key nodes (Figure S4). Transcription factor enrichment analysis for these overlapping proteins further identified many regulators related to immune response, inflammatory regulation, and metabolic homeostasis (e.g., NFKB1, RELA, STAT3, VDR, FOXO1, and PPARG) (Figure S5 and Table S37).

Furthermore, we constructed proteomic and metabolomic signatures for three sarcopenia traits (Table S38). Higher proteomic and metabolomic scores exhibited greater osteoporosis‐free survival probabilities and were significantly associated with reduced risk of osteoporosis in Cox regressions (Figure S6, Table S39). However, for proteomic and metabolomic signatures of heel BMD, only the metabolomic score of left heel BMD had a significant association with sarcopenia risk (Table S40), which is likely due to the smaller sample size of the population with available heel BMD measurements (Table S41).

Additionally, mediation analyses revealed 571 proteins and 205 metabolic biomarkers that mediate the effects of PRSs for osteoporosis or sarcopenia on the prevalences of either disease. Notably, 16.5% (94/571) of proteins and 35.1% (72/205) of metabolic biomarkers were shared mediators across all four PRS‐disease associations (Figure 4D,E; Tables S42–S49). Multiple mediators shared among these associations are known regulators of metabolic homeostasis, immune function, and inflammatory processes, including proteins such as GDF15, PLAUR, and TNFRSF4, as well as metabolites like GlycA, MUFA_pct, and PUFA_by_MUFA (Figure 4D,E).

### 2.5 Genes Potentially Influencing Both Sarcopenia and Osteoporosis

**FIGURE 5:**
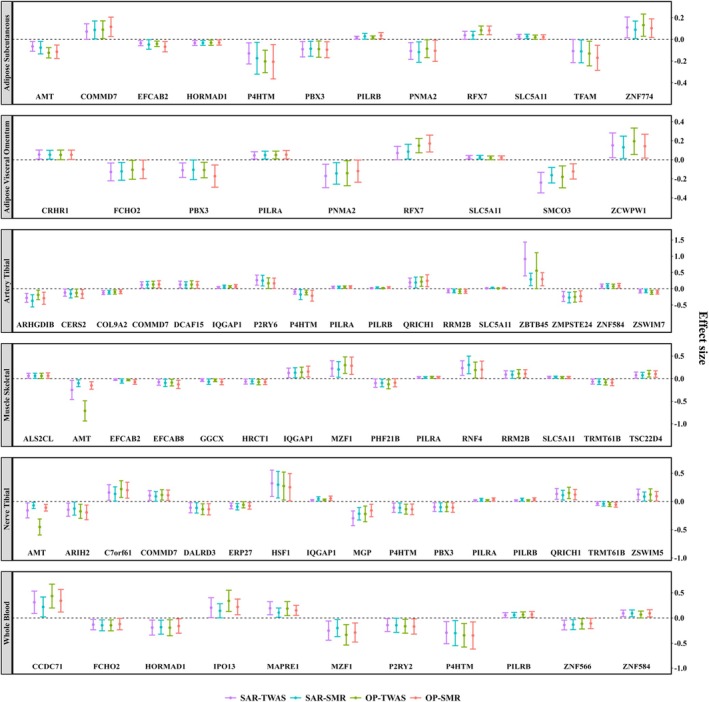
Effect sizes and 95% confidence intervals for the identified genes nominally associated with both sarcopenia and osteoporosis with consistent effect directions in both TWAS and SMR analyses. OP, osteoporosis; SAR, sarcopenia.

Transcriptome‐wide association studies (TWASs) and summary data–based Mendelian randomization (SMR) analyses were conducted to identify genes whose expression levels are related to sarcopenia and osteoporosis risks across 34 human tissues. The analyses revealed dozens of genes showing nominally significant associations with both sarcopenia and osteoporosis in each examined tissue, of which around 70% exhibited consistent effect directions for the two diseases (Tables S50–S52). In tissues likely to influence both sarcopenia and osteoporosis (i.e., adipose subcutaneous, adipose visceral omentum, artery tibial, muscle skeletal, nerve tibial, and whole blood), multiple shared genes were identified by both TWAS and SMR (Figure 5), including some genes (e.g., TFAM, MGP, and COMMD7) with biologically plausible mechanisms relevant to both conditions.

We further performed functional and transcription factor enrichment analyses on the genes identified by TWAS or SMR that were nominally associated with both diseases in consistent effect directions. Functional enrichment analysis revealed multiple metabolic and immune‐related pathways in many analyzed tissues (Tables S53 and S54). Transcription factor enrichment analysis also identified a number of immune‐related transcription factors, such as the RFX complex (RFXANK, RFXAP, and RFX5), enriched in most analyzed tissues (Table S55). However, after applying multiple testing correction, no single gene remained significantly associated with both diseases simultaneously. Accordingly, these nominal associations are presented as exploratory findings that warrant future validation.

### 2.6 Modifiable Factors Exhibiting Similar Effect Patterns on Sarcopenia and Osteoporosis

**FIGURE 6:**
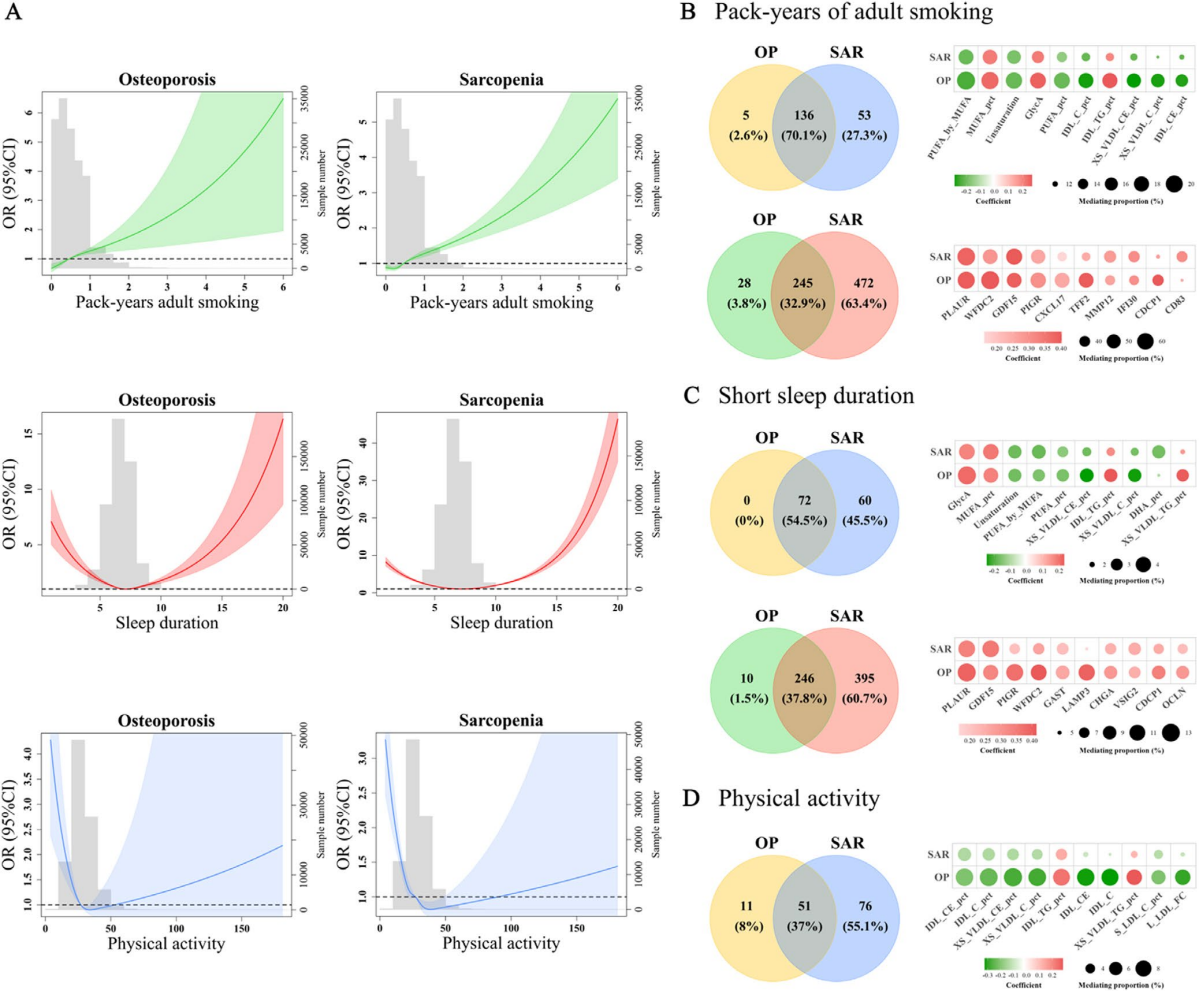
Associations of modifiable factors with sarcopenia and osteoporosis risks. (A) Exposure–response curves depicting odds ratios and 95% confidence intervals for disease risks against modifiable factor levels under restricted cubic spline regressions. Histograms display sample distributions of each factor. (B–D) Mediators in the associations of modifiable factors with sarcopenia and osteoporosis risks. (B) Metabolites (top) and proteins (bottom) for pack‐years of adult smoking. (C) Metabolites (top) and proteins (bottom) for short sleep duration. (D) Metabolites for physical activity (below the thresholds). Venn diagrams show the counts of significant mediators. Bubble plots display the top 10 significant mediators with the largest sum of mediating proportions across the two diseases, with colors indicating effect sizes of standardized mediators on diseases' risks. OP, osteoporosis; SAR, sarcopenia.

Restricted cubic spline regressions were employed to examine the relationships between modifiable factors (including BMI, pack‐years of adult smoking, sleep duration, physical activity, and dietary score) and prevalences of sarcopenia and osteoporosis. The analyses showed that the exposure–response curves for pack‐years of adult smoking, sleep duration, and physical activity had similar patterns between the two diseases (Figure 6A; Figure S7). Specifically, higher pack‐years of adult smoking were monotonically associated with increased prevalences of the two diseases. Sleep duration exhibited U‐shaped relationships with both diseases. Increased physical activity level initially associated with reduced disease prevalences, but the associations became insignificant beyond certain physical activity levels (Table S56). Given these findings, we conducted further stratified analyses for sleep duration and physical activity. Using sleep duration of 6–8 h as the reference, both short sleep duration (< 6 h) and long sleep duration (> 8 h) were associated with increased risks of sarcopenia and osteoporosis. For physical activity, we used the nadir points of the exposure–response curves as stratification thresholds (34.2 milli‐gravity for osteoporosis and 38.8 milli‐gravity for sarcopenia). In participants with physical activity below the thresholds, increased physical activity levels were significantly associated with lower risks of both diseases. However, no significant associations were observed in participants with physical activity above the thresholds (Table S56).

We further investigated protein and metabolite mediators for modifiable factors that demonstrated significant associations with both diseases in subpopulations with available proteomic or metabolomic data. Specifically, we performed proteomic and metabolomic mediation analyses for pack‐years of adult smoking and short sleep duration, as well as metabolomic mediation analyses for physical activity (in participants with physical activity below the thresholds) (Table S56). The analyses revealed numerous mediating proteins and metabolic biomarkers underlying these associations, with substantial overlap (around 30.5%–73%) in mediators between sarcopenia and osteoporosis (Figure 6B–D; Tables S57 and S58).

## 3 Discussion

In this study, we conducted an integrated multi‐omics analysis to systematically investigate the bidirectional relationship and shared biological mechanisms between sarcopenia and osteoporosis using UK Biobank data, in order to advance understanding of muscle‐bone interdependence.

### 3.1 Sarcopenia and Osteoporosis Exhibit Bidirectional Risk Association

Our analyses discovered a bidirectional association between sarcopenia and osteoporosis. Increased muscle function (hand grip strength and usual walking pace) was associated with a reduced risk of osteoporosis, while higher heel BMD was linked to a lower risk of sarcopenia. This reciprocal association has also been revealed in some existing epidemiological studies (Rikkonen et al. 2012; McGrath et al. 2017; Yu et al. 2022). Additionally, we observed a U‐shaped relationship between muscle mass index (ALM/height^2^) and osteoporosis, which was not previously reported in the literature. While most existing studies indicated that muscle growth promotes bone formation through muscle‐derived growth factors and mechanical loading from muscle contractions (Tagliaferri et al. 2015; Ferretti et al. 2003; Brotto and Bonewald 2015), we postulate that our observed adverse effect of excessive muscle growth on osteoporosis is potentially due to overtraining and excessive mechanical loading. Particularly, intense training under conditions of low energy availability suppresses osteocyte metabolism and accelerates bone resorption (Barrack et al. 2014). In females, exercise‐induced amenorrhea and decreased estrogen levels further elevate the risk of bone loss and fractures (Nattiv et al. 2007). Additionally, the osteogenic response to mechanical loading has been found to exhibit signal saturation, where continual increases in skeletal loading eventually lead to diminished bone adaptation (Hart et al. 2017). Prolonged muscle fatigue, irregular loading patterns, and abnormal stress distribution may contribute to cumulative skeletal fatigue and microdamage (Milgrom et al. 2007; Clansey et al. 2012; Herman et al. 2010). Further studies remain necessary to validate this U‐shaped relationship between muscle mass index and osteoporosis as well as its underlying mechanisms.

We further revealed sex‐ and age‐specific effects of sarcopenia traits on osteoporosis risk. Notably, ALM/height^2^ and usual walking pace showed stronger effects on osteoporosis risk in males, and all three sarcopenia traits had larger effects in younger adults. These findings align with some prior epidemiological observations on the associations of sarcopenia traits with osteoporosis or BMD across different sex and age groups (Ho‐Pham et al. 2014; Lee and Shin 2021; Moon 2014). Furthermore, our BMI‐stratified analyses indicated that compared to individuals with higher BMI, those with lower BMI showed stronger effects of ALM/height^2^ but weaker effects of usual walking pace on osteoporosis risk. The greater influence of muscle mass on BMD in individuals with lower BMI has also been observed in an epidemiological study with type 2 diabetes mellitus populations (Pan and Xu 2022). Notably, we also found additive interactions among sarcopenia traits where a concurrent decline in both muscle function indicators further amplified their combined detrimental impact on osteoporosis risk, implying that muscle function may exert greater influence on osteoporosis risk than muscle mass.

### 3.2 Proteins and Metabolites May Mediate the Effect of Muscle on Osteoporosis

This study identified a number of proteins and metabolic biomarkers that mediate the effect of sarcopenia traits on osteoporosis. Many mediating proteins and metabolic biomarkers are involved in immune regulation and inflammatory responses, including proteins such as LGALS9 (Galectin‐9) and LILRA5 (Leukocyte immunoglobulin‐like receptor subfamily A member 5) as well as metabolites like Omega_3_pct (Omega‐3 fatty acids to total fatty acids percentage) and PUFA_pct (polyunsaturated fatty acids to total fatty acids percentage). We also found that these protein mediators are predominantly localized in myeloid cells, endothelial cells, and stromal cells, which are well‐established key regulators of inflammatory responses (Shi et al. 2018; Pober and Sessa 2007; Farr et al. 2016). Existing evidence indicates that sarcopenia may influence immune responses and perpetuate chronic inflammatory states through multiple mechanisms. For instance, decline in muscle mass and function may reduce secretion of myokines, subsequently affecting immune regulation and promoting inflammatory responses (Wang et al. 2024; Nelke et al. 2019). Sarcopenic muscle may also accompany intramuscular fat infiltration, triggering lipotoxic effects and enhancing pro‐inflammatory cytokine production (Li et al. 2022). Notably, chronic inflammation has also been established as a significant contributor to osteoporosis pathogenesis (Clowes et al. 2005). Therefore, together with existing evidence, our findings imply that sarcopenia might contribute to osteoporosis by promoting immune dysregulation and chronic inflammation.

### 3.3 Shared Genetic Architecture and Circulating Biomarkers of Sarcopenia and Osteoporosis

Through LDSC regression, we discovered a positive genetic correlation between sarcopenia and osteoporosis. LAVA method further identified local genetic correlations between the two diseases in 12 genomic regions, including 10 regions showing positive correlations. Furthermore, we found positive associations between sarcopenia PRS and osteoporosis, as well as between osteoporosis PRS and sarcopenia, implying that shared genetic architecture may underlie the two diseases. Consistently, a previous genetic analysis also reported positive genetic correlations between lean mass and total body BMD, as well as negative genetic correlations between lean mass and osteoporosis (Liu, Chen, et al. 2025). Our findings provide further evidence supporting the genetic relationship between sarcopenia and osteoporosis risks.

Furthermore, among the proteins associated with either sarcopenia or osteoporosis, we found that around 31.3% were linked to both diseases with approximately 99.6% concordance in effect directions. These shared proteins are predominantly localized in immune and lymphoid tissues, and significantly enriched in many pathways and transcription factors related to immunity, inflammation, and metabolic regulation. For instance, three of our identified pathways (Th17 cell differentiation pathway, IL‐17 signaling pathway, and NF‐κB signaling pathway) are closely linked to sarcopenia and osteoporosis through inflammatory processes. Th17 (T helper 17) cells, a pro‐inflammatory T cell subset, can activate the NF‐κB signaling pathway through secretion of inflammatory cytokines, particularly IL‐17 (interleukin‐17) (Zhu et al. 2020). NF‐κB (nuclear factor‐κB) represents a family of inducible transcription factors that regulates numerous genes involved in immune and inflammatory responses (Liu et al. 2017). The activation of the NF‐κB pathway contributes to muscle protein breakdown, thereby promoting muscle atrophy (Liu, Wang, Liu, et al. 2024). In bone tissue, NF‐κB activation also enhances bone resorption by stimulating osteoclast differentiation and activity (Abu‐Amer 2013). Several core transcription factors of the NF‐κB signaling pathway, such as NFKB1 (NF‐κB subunit 1), RELA (NF‐κB subunit p65), and REL (NF‐κB subunit c‐Rel) (Cartwright et al. 2016; Campbell et al. 2004), were also identified in our transcription factor enrichment analysis. Additionally, multiple identified transcription factors, such as FOXO1 (forkhead box protein O1), SIRT1 (sirtuin 1), and VDR (vitamin D receptor), have established roles in both sarcopenia and osteoporosis pathogenesis. For instance, FOXO1 and SIRT1 are critical metabolic regulators influencing muscle protein degradation and modulating the balance between bone formation and absorption (Rached et al. 2010; Sin et al. 2015; Lee and Goldberg 2013; Chen et al. 2021). VDR, as a key transcriptional regulator of calcium‐phosphate balance, also plays a crucial role in the regulation of bone mass and secretion of osteotropic hormones (Nakamichi et al. 2017), as well as the maintenance of muscle health by regulating proteolysis, mitochondrial function, and cellular senescence (Bollen et al. 2022).

We also observed around 71% overlap (all showing consistent effect directions) between metabolites associated with sarcopenia and osteoporosis. Many of these metabolites are implicated in metabolic regulation and inflammatory pathways. For instance, omega‐3 polyunsaturated fatty acids such as docosahexaenoic acid (DHA) were identified as shared biomarkers. Chronic inflammation is known to play a pivotal role in the pathogenesis of both sarcopenia and osteoporosis (Cheng et al. 2025; Livshits and Kalinkovich 2022). Emerging evidence suggests that omega‐3 fatty acids may exert protective effects against osteoporosis and sarcopenia by their anti‐inflammatory properties (Huang et al. 2020; Rozner et al. 2020).

Our study also revealed substantial overlap in mediating proteins and metabolites across four PRS‐disease associations, with 16.5% of proteins and 35.1% of metabolites serving as shared mediators. Notably, multiple mediators shared among these associations are known regulators of metabolic homeostasis, immune function, and inflammatory processes. For instance, GDF15 (growth/differentiation factor 15) is involved in stress response and metabolic regulation (Johann et al. 2021); TNFRSF4 (tumor necrosis factor receptor superfamily member 4) is an immune‐modulating molecule expressed on T cells (Schreiber et al. 2012); GlycA (glycoprotein acetyls) represents well‐established inflammatory biomarkers (Connelly et al. 2017). These findings suggest that shared circulating biomarkers involved in these biological processes potentially mediate genetic effects on both diseases.

On the other hand, proteomic and metabolomic scores for sarcopenia traits were found to be associated with osteoporosis. However, our assessments relied on internal rather than external validation. Moreover, these scores do not provide biological interpretability at the individual protein or metabolite level. Consequently, our findings require further validation and mechanistic exploration.

### 3.4 Genes Potentially Influencing Both Sarcopenia and Osteoporosis

Our analysis identified a number of genes nominally associated with both sarcopenia and osteoporosis in consistence effect direction. Among them, multiple genes (i.e., TFAM, COMMD7, and MGP) have plausible biological links to both conditions. For example, TFAM (mitochondrial transcription factor A) is a critical regulator of mitochondrial DNA maintenance (Alam et al. 2003). Deficiency in TFAM may lead to mitochondrial dysfunction, which has been identified as a contributor skeletal muscle atrophy (Chen et al. 2023) and impair osteogenesis (Zheng et al. 2020); MGP (matrix Gla protein) encodes a vitamin K‐dependent protein that functions as a calcification inhibitor in soft tissues, which is also known as a crucial regulator of bone metabolic homeostasis (Julien et al. 2009) and demonstrate upregulation during myogenesis (Ahmad et al. 2017); COMMD7 (copper metabolism Murr1 domain containing 7) is involved in NF‐κB signaling activation, which is a well‐known inflammatory pathway contributing to sarcopenia and osteoporosis (Zheng et al. 2019). Functional enrichment analysis of the shared genes linked to both diseases also revealed multiple pathways (e.g., type I diabetes mellitus, Th1 and Th2 cell differentiation, and MHC [major histocompatibility complex] class II protein complex assembly), suggesting their potential involvement in disease pathogenesis. For instance, type I diabetes mellitus has been associated with musculoskeletal dysfunction, likely through insulin deficiency, hyperglycemia, and dysregulation of bone‐ and muscle‐derived cytokines (Weber and Schwartz 2016; Travis et al. 2022); Th1 (T helper 1) and Th2 (T helper2) cells have been found to affect bone remodeling and muscle repair processes by participating in inflammatory response (Tidball and Villalta 2010; Zhang et al. 2022); MHC class II proteins play essential roles in adaptive immune responses and T cell activation (Neefjes et al. 2011). Activated T cells can modulate bone health and regulate bone remodeling through the secretion of various cytokines such as RANKL (Pacifici 2016; Srivastava et al. 2018). Regulatory T cells have also been shown to promote muscle regeneration in both acute and chronic injury (Cho et al. 2019). Transcription factor enrichment analysis further identified the RFX complex (comprising RFXANK, RFXAP, and RFX5) in many analyzed tissues, which collectively regulate the transcription of MHC class II (Villard et al. 2000), suggesting a potential immunoregulatory link to musculoskeletal degeneration.

However, it is also important to note that under adjustment for multiple comparisons, no gene achieved significance for the associations with both diseases simultaneously in our TWAS and SMR analyses. The nominal associations reported here are thus for exploratory purposes. Therefore, these findings should be interpreted cautiously and await confirmation through further validation.

### 3.5 Lifestyle Factors Exhibiting Similar Effect Patterns on Sarcopenia and Osteoporosis

This study revealed that lifestyle factors, including pack‐years of adult smoking, sleep duration, and physical activity, exhibited similar patterns of influence on both diseases. Our findings confirmed the detrimental effects of smoking on both sarcopenia and osteoporosis. Existing evidence demonstrates that smoking promotes chronic inflammation, oxidative stress, and mitochondrial dysfunction, collectively contributing to muscle atrophy and functional decline (Degens et al. 2015). Concurrently, smoking disrupts bone metabolic homeostasis, leading to reduced bone mass (Yoon et al. 2012).

Regarding sleep duration, we observed that both insufficient and excessive sleep elevated the risk of sarcopenia and osteoporosis, consistent with multiple epidemiological studies (Li et al. 2023; Rubio‐Arias et al. 2019; Ochs‐Balcom et al. 2020; Kim et al. 2014; Saint Martin et al. 2016). Current evidence suggests that sleep deprivation disrupts hormonal regulation and promotes chronic low‐grade inflammation, which may impair muscle protein synthesis and inhibit osteoblast proliferation (Piovezan et al. 2015; Mullington et al. 2010; Kuriyama et al. 2017). Conversely, prolonged sleep duration appears to facilitate sarcopenia and osteoporosis through reduced physical activity levels, exacerbated inflammation, and metabolic dysregulation (Li et al. 2023; Patel et al. 2009; Chen et al. 2014).

Our study revealed that increased physical activity (below certain thresholds) is associated with reduced prevalences of both diseases. This finding aligns with current clinical guidelines that recommend physical activity as a primary treatment of sarcopenia (Dent et al. 2018). Mechanistically, prior research indicated that physical inactivity promotes visceral adiposity, triggering chronic inflammation and metabolic dysregulation that compromise musculoskeletal health (Pedersen 2009). Physical activity has also been found to enhance mechanical loading on bones, thereby promoting bone remodeling and improving bone mineral density (Guadalupe‐Grau et al. 2009; Nowak and Ogurkowska 2024). However, while physical activity generally benefits musculoskeletal health, excessive exercise may negate these protective effects due to induced systemic inflammation and energy deficiency (Smith 2000; Angelidi et al. 2024).

For these lifestyle factors, we also observed substantial overlap (> 30%) in protein and metabolite mediators between sarcopenia and osteoporosis, suggesting that these lifestyle factors might affect the two diseases through some similar biological mechanisms. Specifically, for smoking and short sleep duration, many overlapping mediators exhibit immunomodulatory and inflammatory properties, such as inflammatory biomarker PLAUR (urokinase plasminogen activator receptor) (Thunø et al. 2009), as well as unsaturated fatty acids indices PUFA_by_MUFA (polyunsaturated fatty acids to monounsaturated fatty acids ratio) and MUFA_pct (monounsaturated fatty acids to total fatty acids percentage), which are closely linked to oxidative stress and chronic inflammation (Morgese et al. 2021; van Dijk et al. 2009; Montserrat‐de la Paz et al. 2023). These findings support existing evidence that both tobacco exposure and sleep deficiency upregulate pro‐inflammatory cytokines and promote chronic inflammation (Mullington et al. 2010; Khan et al. 2020; Arnson et al. 2010), a key pathological mechanism common to both sarcopenia and osteoporosis (Cheng et al. 2025; Livshits and Kalinkovich 2022). Furthermore, for smoking and short sleep duration, we also identified multiple gastrointestinal‐related mediators common to both diseases. For instance, PIGR (polymeric immunoglobulin receptor) and TFF2 (trefoil factor 2) support intestinal health through mucosal immune protection and epithelial repair mechanisms, respectively (Kaetzel 2005; Taupin and Podolsky 2003); and GAST (gastrin) regulates gastric acid secretion and gastric mucosa growth (Rozengurt and Walsh 2001). These observations align with some established evidence, which indicates that smoking compromises intestinal epithelial barrier function, increasing susceptibility to gut inflammation and microbial dysbiosis (Fricker et al. 2018; Chen, Zeng, et al. 2024), while sleep deficiency exacerbates digestive disorders and disrupts gut microbiota homeostasis (Orr et al. 2020; Gao et al. 2019). Notably, gastrointestinal dysfunction and gut dysbiosis have been implicated in musculoskeletal deterioration through chronic inflammation and impaired nutrient absorption (Ticinesi et al. 2019; Nishikawa et al. 2021; Chen et al. 2017; Ohlsson and Sjögren 2015). For physical activity, the mediating metabolic biomarkers shared between sarcopenia and osteoporosis are predominantly involved in lipid metabolism. Existing epidemiological studies have also indicated an association between dysregulated lipid metabolism and compromised musculoskeletal health (Liu, Yang, et al. 2025; Zhang et al. 2020; Bi et al. 2024; Liu, Wang, Shen, et al. 2024). Dyslipidemia is found to accelerate muscle degeneration by triggering insulin resistance and inflammation (Lipina and Hundal 2017; Schrauwen 2007), as well as impair bone homeostasis and vascularization by inducing oxidative stress and marrow adipogenesis (Anagnostis et al. 2022; Xiao et al. 2024).

Collectively, for the adverse effects of smoking, sleep deficiency, and low physical activity on sarcopenia and osteoporosis, our results implied that circulating biomarkers involving biological processes, such as chronic inflammation, gastrointestinal dysfunction, and lipid metabolism, might be shared mediators between the two diseases.

### 3.6 Limitations

In this study, there are still several limitations. First, we used “probable sarcopenia” as sarcopenia diagnosis in our primary analysis based solely on grip strength, which may introduce misclassification bias. On the other hand, the precise timing of sarcopenia onset is unknown under this sarcopenia definition. Therefore, selection bias may occur in the longitudinal analyses with sarcopenia outcome, because individuals who developed sarcopenia between baseline and the imaging visit might have been excluded due to disease progression or death. Moreover, although we adjusted for the time interval between baseline and imaging visit to account for potential confounding, the linear adjustment may not fully capture the dynamic trajectory of sarcopenia risk over time.

Second, we utilized heel BMD as an osteoporosis trait in our primary analysis, due to the lack of baseline BMD measurements at other skeletal sites in the UK Biobank. However, since heel quantitative ultrasound is not universally considered the gold standard for diagnosing osteoporosis, these findings should be interpreted with caution and validated in future studies using BMD measured at the femoral neck or lumbar spine.

Third, our mediation analyses did not establish temporal ordering among exposure, mediator, and outcome simultaneously due to data constraints in the UK Biobank. Therefore, the observed mediation effects in this study should be interpreted as exploratory and associative rather than as causal sequential mediation. Future studies incorporating longitudinal mediator assessments and experimental designs are warranted to validate our findings.

Fourth, the multi‐omics findings of this study are based on observational data, in which residual unmeasured confounding may influence our findings—for instance, by potentially inflating the observed overlap in risk‐associated proteins and metabolites between sarcopenia and osteoporosis. Consequently, these findings should be interpreted as exploratory and hypothesis‐generating. Future studies incorporating animal models and cellular experiments are warranted to elucidate the underlying causal mechanisms.

## 4 Conclusion

This study demonstrated that sarcopenia and osteoporosis may act as reciprocal risk factors and potentially have shared biological mechanisms. We identified numerous proteins and metabolites that appear to mediate the influence of muscle on bone health, providing mechanistic insights into this relationship. The discovery of genetic correlation, as well as overlapping risk‐associated genes and circulating biomarkers between sarcopenia and osteoporosis, highlights common pathophysiological pathways underlying both conditions. Lifestyle factors, including smoking, sleep duration, and physical activity, demonstrated similar effect patterns and substantial overlap of mediating proteins/metabolites between both diseases, suggesting potential for joint prevention strategies. These findings underscore the interconnected nature of musculoskeletal health and lay the groundwork for future research aimed at targeting shared biological pathways and developing common interventions for both conditions.

## 5 Methods

### 5.1 Study Population

This study utilized data from the UK Biobank, a large‐scale cohort initiated in 2006, which enrolled over 500,000 participants aged 40–69 years at baseline (Sudlow et al. 2015). The project has collected extensive phenotypic and genotypic data, including demographic profiles, standardized clinical measurements, biological samples, and multimodal imaging. Ethical approval for the UK Biobank study was granted by the North West Multicentre Research Ethics Committee (REC reference: 21/NW/0157). All participants provided written informed consent prior to enrollment. Researchers accessing UK Biobank data were exempt from additional ethical review under the approved framework.

### 5.2 Sarcopenia Diagnosis and Related Traits

According to the European Working Group on Sarcopenia in Older People (EWGSOP) (Cruz‐Jentoft et al. 2019), muscle mass, muscle strength, and physical function are three key sarcopenia‐related traits, which can be assessed by lean mass, hand grip strength, and usual walking pace, respectively. In this study, we evaluated muscle mass by appendicular lean mass normalized to standing height squared (m^2^) (expressed as ALM/height^2^), where ALM was converted from appendicular fat‐free mass (sum of limb segments) using the formula: ALM (kg) = 0.958 × [appendicular fat‐free mass (kg)] − 0.166 × *G* − 0.308 (*G* = 0 and 1 for female and male, respectively) (Dodds et al. 2020). Hand grip strength (kg) was calculated as the sum of grip strength of left and right hands, measured using a Jamar J00105 hydraulic handheld dynamometer. Usual walking pace was self‐reported by participants through a questionnaire with three response options, including slow pace (< 3 miles/h), steady average pace (3–4 miles/h), and brisk pace (over 4 miles/h), which were coded as 0, 1, and 2 respectively. In this study, baseline measurements of ALM/height^2^, hand grip strength, and usual walking pace were used as sarcopenia‐related traits unless otherwise specified.

Following the sarcopenia definition from the EWGSOP, “probable sarcopenia” is defined by low grip strength (< 27 kg in males and < 16 kg in females). “Confirmed sarcopenia” is defined by the presence of both low grip strength and low muscle mass (ALM/height^2^ < 7.0 kg/m^2^ in males and < 5.5 kg/m^2^ in females). In the primary analysis of this study, we used probable sarcopenia for sarcopenia diagnosis at baseline and imaging visit, whereas confirmed sarcopenia was used in complementary analyses.

### 5.3 Osteoporosis Diagnosis and Related Traits

Osteoporosis was systematically evaluated by linking the data of all participants to national health registries and primary care records, with cases identified through the International Classification of Diseases Tenth Revision (ICD‐10) codes M80‐M81. Participants were monitored from baseline until first diagnosis of osteoporosis, death, loss to follow‐up, or the end of follow‐up (i.e., December 31, 2022). For osteoporosis‐related traits, baseline measurement of BMD at left and right heels and their corresponding *T*‐scores were used in our primary analysis. Heel BMD was estimated based on the Quantitative Ultrasound Index through the calcaneus. Heel BMD *T*‐scores were calculated from the ultrasound heel BMD measurement and standardized by comparing with sex‐matched healthy reference population. The units of the *T*‐score are the number of standard deviations that the bone density is above or below the standard. Additionally, femoral neck BMD at both sides and lumbar spine (lumbar vertebrae 1–4) BMD measured by dual‐energy X‐ray absorptiometry (DXA) at the imaging visit were used as alternative osteoporosis‐related traits in our complementary analysis.

### 5.4 Olink Proteomics and NMR Metabolomics Data

The UK Biobank Pharma Proteomics Project (UKB‐PPP) performed high‐throughput proteomic profiling on 54,306 plasma samples using the Olink Explore 1536 platform, which employed antibody‐based Proximity Extension Assay to quantify 2923 circulating protein biomarkers across multiple disease domains, including inflammation, oncology, cardiometabolic, and neurological disorders (Table S59).

The UK Biobank quantified 251 metabolomic biomarkers in plasma samples from approximately 280,000 participants using high‐throughput nuclear magnetic resonance metabolomic profiling platform (Table S60). This analysis measured 170 metabolites and 81 composite ratio biomarkers, encompassing a broad spectrum of metabolomic indices of low‐molecular‐weight metabolites (e.g., amino acids, ketone bodies, and glycolysis metabolites), lipidomic profiles of lipoproteins, fatty acids and fatty acid compositions.

All proteomic and metabolomic data used in this study originated from the baseline assessment of the UK Biobank.

### 5.5 Modifiable Variables

Modifiable variables consisted of BMI (kg/m^2^), pack‐years of adult smoking (the average number of pack‐years smoked by an individual each year over their adult lifetime), sleep duration (hours/day), physical activity (the overall level of physical activity measured by accelerometer in milli‐gravity units), and dietary score. The dietary score was calculated based on seven food categories from the UK Biobank, with each assigned a binary score (1 if meeting intake thresholds, 0 otherwise): fruits (≥ 3 servings/day), vegetables (≥ 3 servings/day), fish (≥ 2 servings/week), processed meats (≤ 1 serving/week), unprocessed red meats (≤ 1.5 servings/week), whole grains (≥ 3 servings/day), and refined grains (≤ 1.5 servings/day). The total score (range: 0–7) reflected adherence to healthy dietary guidelines, with higher scores indicating better alignment (Lourida et al. 2019). In this study, we used baseline measurements of these modifiable variables.

### 5.6 Mendelian Randomization

Mendelian randomization (MR) enables causal effect estimation in the presence of unmeasured confounding, provided that three core assumptions (i.e., relevance, independence, and exclusion restriction) of valid instrumental variables (IVs) are satisfied. In this study, we performed bidirectional MR analyses to further assess the causal association between sarcopenia and osteoporosis.

In the MR analyses, lumbar spine BMD was used as an osteoporosis‐related trait, for which genome‐wide association study (GWAS) summary statistics were derived from eight cohorts comprising 33,297 participants of European or white ancestry (see Table S61) (Zheng et al. 2015) (GWAS Catalog accession: https://www.ebi.ac.uk/gwas/publications/26367794). For sarcopenia, we analyzed nine sarcopenia‐related traits using GWAS data from approximately 460,000 Europeans in the UK Biobank (MRC‐IEU accession: https://gwas.mrcieu.ac.uk), including whole body fat‐free mass (GWAS ID: ukb‐b‐13354), trunk fat‐free mass (GWAS ID: ukb‐b‐17409), right leg fat‐free mass (GWAS ID: ukb‐b‐12828), left leg fat‐free mass (GWAS ID: ukb‐b‐16099), right arm fat‐free mass (GWAS ID: ukb‐b‐19520), left arm fat‐free mass (GWAS ID: ukb‐b‐19925), right hand grip strength (GWAS ID: ukb‐b‐10215), left hand grip strength (GWAS ID: ukb‐b‐7478), and usual walking pace (GWAS ID: ukb‐b‐4711).

The instrumental variables for each exposure were selected as follows. First, we chose significant single nucleotide polymorphisms (SNPs) with *p* < 5 × 10^−8^ from the exposure GWAS to satisfy the IV relevance assumption. Second, we removed SNPs within the MHC region (chromosome 6: 26–34 Mb) due to complex linkage disequilibrium (LD) patterns. Third, LD clumping was performed to remove correlated SNPs within 5 Mb and correlation *r* ^2^ > 0.01.

The causal effects between sarcopenia and osteoporosis traits were estimated by the inverse variance weighted (IVW) method as the primary analysis, which provides consistent estimates under the three core assumptions of valid IVs (Burgess et al. 2013). As a sensitivity analysis, we additionally employed the MR‐Median method, which remains reliable even when up to 50% of the IVs are invalid and thus is robust to violations of the independence and exclusion restriction assumptions (Bowden et al. 2016). To assess potential reverse causality, we applied MR‐Steiger method (Hemani et al. 2017) to the significant associations (IVW *p* < 0.05 and MR‐Median *p* < 0.05). A correct causal direction with *p* < 0.05 represents a causal direction from the exposure to the outcome. All MR and Steiger filtering analyses were conducted using the “TwoSampleMR” R package.

### 5.7 Analysis of Single‐Cell Transcriptomic Data

This study utilized scRNA‐seq data from the human skeletal muscle aging atlas established by Kedlian et al. (2024), comprising 90,902 cells from intercostal muscles of 17 donors (8 young adults aged 20–40 years and 9 older adults aged 60–75 years). All single‐cell data processing and visualization were performed using R software. Quality control and normalization were conducted with the “Seurat” R package, applying the following filtering criteria: (1) cells containing 300–10,000 detected genes; (2) cells with > 600 unique molecular identifier counts; (3) cells with < 10% mitochondrial gene content; and (4) cells with < 1% hemoglobin gene content.

After performing log‐normalization and scaling of data, we conducted principal component analysis using 2000 highly variable genes and retained the top 41 principal components that explained more than 90% variance. Batch effects across samples were corrected for using the “Harmony” R package. For cell clustering, we chose optimal resolution from 0.1 to 2 by an increment of 0.1 using the “FindClusters” function in Seurat and selected resolution = 1 for optimal separation. Cell clusters were visualized via uniform manifold approximation and projection (UMAP) using the “RunUMAP” function, and annotated into major types based on established marker genes (Kedlian et al. 2024). For proteins mediating the associations between sarcopenia traits and osteoporosis risk, we investigated their gene expression profiles across various cell types in muscle tissue based on scRNA‐seq data. Cell type enrichment was determined when the average expression ratio between target and nontarget cell types was larger than four (Oh et al. 2023).

### 5.8 Proteomic and Metabolomic Scores

In the analyses of proteomic and metabolomic scores, the dataset was randomly split into training set and testing set by the ratio of 3:7 for model development and inference, respectively. For model development, we built up one‐dimensional convolutional neural networks (1D‐CNN) to construct proteomic and metabolomic scores for sarcopenia traits and osteoporosis traits, respectively. The 1D‐CNN models used expression levels of 2923 proteins or 251 metabolites as input and sarcopenia or osteoporosis traits as output. The 1D‐CNN architecture consisted of three convolutional layers (kernel size = 2, channels = 32, stride = 1), three pooling layers (pooling window size = 2), and two fully connected layers with L2 regularization and dropout (0.3), followed by a single linear neuron for regression output. The models were trained by Adam optimizer (learning rate = 1e−4) with early stopping (patience = 10). Mean squared error and Pearson correlation were used as performance metrics for model evaluation. The models were constructed using Python 3.9.

### 5.9 GWAS Analyses

We conducted GWASs for osteoporosis and sarcopenia based on 409,373 individuals of Caucasian ancestry from the UK Biobank. For sarcopenia, we applied the “probable sarcopenia” definition from EWGSOP for baseline diagnosis. For osteoporosis, we used ICD‐10 codes M80‐M81 to identify osteoporosis cases by the end of follow‐up. We performed quality control of genetic variants using PLINK2 based on the following exclusion criteria: missingness rates > 0.05, minor allele frequency < 0.001, Hardy–Weinberg equilibrium test *p* < 1 × 10^−6^, and imputation information scores < 0.8. GWASs were performed based on the generalized mixed linear model using the fastGWA‐GLMM method (Jiang et al. 2021) implemented in the GCTA software for autosomal genome‐wide scanning. For both diseases, the adjusted covariates include age, genetic sex, genotype measurement batch (BiLEVE or Axiom array), and the top 20 genetic principal components.

### 5.10 TWAS and SMR Analyses

TWASs for osteoporosis and sarcopenia risks were performed using the S‐PrediXcan (Barbeira et al. 2018) method, based on the corresponding GWAS data as well as MASHR‐based transcriptome prediction models and pre‐computed covariance matrices derived from expression quantitative trait locus (eQTL) data in version 8 (v8) of the Genotype‐Tissue Expression (GTEx) project (GTEx Consortium 2013). The analyses were performed using the “SPrediXcan” software in Python 3.9. Nominally significant associations were identified based on TWAS *p* < 0.05.

We also identified genes associated with osteoporosis and sarcopenia risks based on GWAS data and GTEx v8 eQTL data by the SMR method (Zhu et al. 2016) implemented on the SMR Portal (https://yanglab.westlake.edu.cn/smr‐portal/task/create), where eQTLs with *p* < 5 × 10^−8^ were used for the SMR test. To further test whether there was a single variant associated with both trait and gene expression, the heterogeneity in dependent instruments (HEIDI) test (Zhu et al. 2016) was performed using eQTLs with *p* < 1.57 × 10^−3^ (a default threshold for HEIDI test). Nominally significant associations were identified based on SMR *p* < 0.05 and HEIDI *p* > 0.05.

### 5.11 Heritability and Genetic Correlation Estimation

Heritability of osteoporosis and sarcopenia along with their genetic correlation across genomes were estimated based on their GWAS results by LDSC regression (Bulik‐Sullivan et al. 2015). Analysis was performed using the “ldsc” software in Python 3.9, leveraging SNPs in the HapMap 3 reference panel, and pre‐computed LD scores and regression weights based on European data from the 1000 Genomes Project (https://alkesgroup.broadinstitute.org/LDSCORE/eur_w_ld_ch).

Local heritability and genetic correlations for osteoporosis and sarcopenia within 2495 genomic loci were estimated by the LAVA method (Werme et al. 2022) using the “LAVA” R package. We used the European panel from 1000 Genomes Project Phase 3 for LD estimation, and used 2495 predefined genomic loci generated by LAVA, which partitions the genome into approximately equal‐sized blocks (1 Mb) while minimizing LD between blocks based on the same European reference panel. Sample overlap between the GWASs of osteoporosis and sarcopenia was accounted for with the estimated intercept term of LDSC regression.

### 5.12 PRS Derivation

The PRSs of osteoporosis risk and sarcopenia risk were derived for Caucasian individuals in the UK Biobank using independent SNPs with corresponding GWAS *p* < 0.05, where SNPs with correlation *r* ^2^ > 0.01 within 5 Mb were excluded by LD clumping. The PRS was then calculated using the ‐score option in PLINK2 as the sum of the count of risk alleles multiplied by the corresponding effect size from the GWAS across the selected SNPs.

### 5.13 Tissue Mapping and Subcellular Localization of Identified Proteins

To infer the possible tissue origins of the identified proteins linked to sarcopenia and osteoporosis in consistent direction, we performed tissue enrichment analyses using the established tissue enrichment profiles from previous studies (Oh et al. 2023; Carrasco‐Zanini et al. 2024) based on GTEx and Human Protein Atlas (HPA) databases (Uhlén et al. 2015). Additionally, we performed subcellular localization analysis to delineate the cellular distribution patterns of these proteins, classifying them into secreted, plasma membrane‐associated, or intracellular proteins (Goeminne et al. 2025).

### 5.14 Bioinformatics Analyses of Identified Proteins and Genes

For the identified proteins and genes associated with sarcopenia and osteoporosis in consistent direction, we performed Kyoto Encyclopedia of Genes and Genomes (KEGG) and Gene Ontology (GO) enrichment analyses using the “ClusterProfiler” R package to identify relevant pathways and biological processes. Enrichment significance was determined using Benjamini–Hochberg false discovery rate (FDR) correction (FDR < 0.05). To characterize functional interplay among the proteins, we constructed a protein–protein interaction (PPI) network using the STRING database (https://www.string‐db.org) under an interaction score threshold of 0.9 (highest confidence). Additionally, we conducted transcription factor enrichment analysis via the TRRUST database (https://www.grnpedia.org/trrust/) to identify potential upstream transcriptional regulators of the identified proteins.

### 5.15 Statistical Analyses

In this study, we analyze the associations of various exposures (i.e., sarcopenia traits and diagnosis, osteoporosis traits and diagnosis, proteins and metabolites, proteomic and metabolomic scores, PRSs and modifiable factors) with osteoporosis risk or sarcopenia risk, respectively, by longitudinal analyses and/or cross‐sectional analyses. In longitudinal analyses with osteoporosis risk, Cox regression models were constructed. In longitudinal analyses with sarcopenia risk, due to the uncertain onset time of sarcopenia defined in this study, we applied logistic regressions using sarcopenia diagnosis at the imaging visit (2014 onward) and further adjusting for the time difference between the imaging visit and the initial assessment visit (baseline visit, 2006–2010). In cross‐sectional analyses, logistic regressions were employed for both osteoporosis prevalence or sarcopenia prevalence at baseline. Unless otherwise specified, the exposures were measured at baseline, and the adjusted covariates include sex, age, ethnicity, and BMI in both longitudinal and cross‐sectional analyses. In this study, restricted cubic spline regressions were also constructed using “rms” R package to visualize the exposure–response curves for continuous exposures, where the optimal numbers of knots were chosen from three to five based on the Akaike information criterion, and median values of the exposures were used as reference when computing OR or HR in the exposure–response curves. For continuous exposures, including ALM/height^2^, hand grip strength, and proteomic and metabolomic scores, we also created categorical variables by stratifying them into tertiles (i.e., low, medium, and high). Kaplan–Meier analysis was then used to compare survival curves across different levels of these exposures.

Specifically, to evaluate the effect of sarcopenia on osteoporosis risk, longitudinal analyses were first conducted to examine the association for each of the three sarcopenia traits (treated as continuous variables) with osteoporosis risk. Moreover, we conducted additional longitudinal analyses for categorical usual walking pace, and performed restricted cubic spline regressions for ALM/height^2^ and hand grip strength to visualize the exposure–response curves. Further, we estimated the effects of multiplicative interactions between the covariates (sex, age, BMI) and continuous sarcopenia traits on osteoporosis risk, respectively, where age was categorized into non‐elderly (< 60 years) and elderly (≥ 60 years) groups, and BMI was classified as non‐overweight (< 25 kg/m^2^) and overweight (≥ 25 kg/m^2^). If significant multiplicative interaction effects were found, we performed stratified analysis to estimate the effects of sarcopenia traits on osteoporosis risk within different groups of the corresponding covariates. For additive interactions between each pair of categorical sarcopenia traits, we calculated the relative excess risk due to interaction (RERI) using the “InteractionR” R package. Further, we estimated the PAFs for osteoporosis risk by longitudinal analyses with categorical sarcopenia traits. We also examined the longitudinal associations of sarcopenia traits at the imaging visit (as sensitivity analysis) and sarcopenia diagnosis at baseline with osteoporosis risk. Cross‐sectional analyses were also conducted to investigate the effects of sarcopenia traits and sarcopenia diagnosis on osteoporosis prevalence at baseline. Similar analyses were also performed to assess the influence of osteoporosis on sarcopenia risk. Osteoporosis traits include left and right heels BMD and *T*‐scores, as well as categorized *T*‐scores consisting of osteoporosis (*T*‐score ≤ −2.5), osteopenia (−2.5 < *T*‐score < −1.0), and normal (*T*‐score ≥ −1.0) according to WHO criteria (Eriksen 2012). We conducted longitudinal and cross‐sectional analyses for associations of osteoporosis traits and diagnosis with sarcopenia risk, restricted cubic spline regressions for exposure–response curves, evaluation of multiplicative interaction between heel BMD *T* scores and covariates, testing for additive interactions between left and right categorized *T*‐scores, and estimation of PAF of heel BMD *T*‐scores for sarcopenia risk.

Mediation analyses were performed to examine the potential mediating roles of proteins and metabolites in the longitudinal associations between sarcopenia‐related traits and osteoporosis risk. The effects of sarcopenia traits on mediators were estimated using multiple linear regression models at baseline, adjusted for covariates including sex, age, ethnicity, and BMI. The effects of mediators on osteoporosis risk were obtained by longitudinal analyses. The 95% confidence intervals (CIs) of mediation effects were calculated based on the percentile‐based method using nonparametric bootstrap with 1000 resamples. To explore shared biological mechanisms between sarcopenia and osteoporosis, we conducted cross‐sectional analyses to evaluate the associations of PRSs for both diseases, circulating biomarkers, and modifiable factors with disease prevalences, respectively. Further mediation analyses were applied to investigate protein and metabolite mediators in the associations of PRSs and modifiable factors with both diseases. Note that, given the uncertain onset timing of sarcopenia, we conducted cross‐sectional analyses rather than longitudinal analyses to ensure the consistencies in the analyses for the two diseases when investigating their shared mechanisms.

The statistical analyses were performed using R software (version 4.4.1) unless otherwise noted. For the analyses involving proteomic/metabolomic associations and mediations, TWAS and SMR, we applied the Benjamini–Hochberg procedure to control the false discovery rate (FDR). In these cases, statistical significance was defined as FDR < 0.05 unless otherwise specified. For all other analyses, statistical significance was determined as *p* < 0.05.

## Author Contributions

S.X., S.W., X.Z., and J.Z. were responsible for data analysis and interpretation, and contributed to drafting the manuscript. Q.D., Y.L., K.W., and P.C. contributed to data acquisition. C.D., Y.Z., and G.R. contributed to the conception and design of the study, and revised the manuscript.

## Funding

This work was supported by Science and Technology Projects in Guangzhou (2025A04J3955), National Natural Science Foundation of China (82472478), and Clinical Research Hongmian Project of Guangzhou First People's Hospital (HM2025039).

## Conflicts of Interest

The authors declare no conflicts of interest.

## Supporting information


**Figure S1:** Exposure–response relationships between osteoporosis traits and sarcopenia. Exposure–response curves depicting odd ratios and 95% confidence intervals for sarcopenia risk against osteoporosis traits under restricted cubic spline regressions. Histograms display sample distributions of each trait. BMD, bone mineral density.
**Figure S2:** Marker gene expressions of different cell types in skeletal muscle. Average expression represents the average of standardized gene expression levels within each cell type. Percent expression denotes the percentage of cells expressing the given gene within each cell type.
**Figure S3:** Scatter plots of SNP effect sizes for sarcopenia risk (*x*‐axis) and osteoporosis risk (*y*‐axis) in genomic regions with significant local genetic correlation. Red and blue dots denote SNPs significantly associated with both traits (*p* < 0.05) in consistent and opposite directions, respectively. Gray dots represent other SNPs in these regions. The slope of dashed line indicates the estimated local genetic correlation (*r*). *r* and *p* denote the local genetic correlation estimate and corresponding *p* value, respectively.
**Figure S4:** Protein–protein interaction (PPI) network for proteins associated with both osteoporosis and sarcopenia in consistent effect directions. The PPI network has a clustering coefficient of 0.318, containing 496 nodes and 353 edges (expected number of edges: 67, enrichment *p* value < 1 × 10^−16^).
**Figure S5:** Transcription factors enrichment profile of proteins associated with both osteoporosis risk and sarcopenia risk.
**Figure S6:** Associations of proteomic and metabolomic signatures for sarcopenia traits with osteoporosis risk. (A, B) Survival curves for osteoporosis onset stratified by (A) proteomic and (B) metabolomic scores for sarcopenia traits. (C) Forest plot of hazard ratios and 95% confidence intervals for the associations of proteomic and metabolomic scores of sarcopenia traits with osteoporosis risk.
**Figure S7:** Exposure–response relationships between modifiable factors (body mass index and dietary score) and sarcopenia. Exposure–response curves depicting odds ratios and 95% confidence intervals for sarcopenia and osteoporosis risks against modifiable factors under restricted cubic spline regressions. Histograms display sample distributions of BMI and dietary score. BMI, body mass index.

**Table S1:** Longitudinal associations between sarcopenia traits and osteoporosis risk.
**Table S2:** Longitudinal associations of multiplicative interactions between sarcopenia traits and covariates with osteoporosis risk.
**Table S3:** Longitudinal associations between sarcopenia traits and osteoporosis risk stratified by the covariates.
**Table S4:** Longitudinal associations for pairwise combinations of sarcopenia traits with osteoporosis risk.
**Table S5:** Population attributable fractions (PAFs) of sarcopenia traits for osteoporosis risk.
**Table S6:** Longitudinal associations of sarcopenia diagnosis (at baseline) and sarcopenia traits (at imaging visit) with osteoporosis risk.
**Table S7:** Cross‐sectional associations of sarcopenia diagnosis and sarcopenia traits with osteoporosis prevalence.
**Table S8:** Mendelian randomization analyses for the effects of sarcopenia traits on bone mineral density of lumbar spine.
**Table S9:** Longitudinal associations between osteoporosis traits and sarcopenia risk.
**Table S10:** Longitudinal associations of multiplicative interactions between osteoporosis traits and covariates with sarcopenia risk.
**Table S11:** Longitudinal associations for pairwise combinations of osteoporosis traits with sarcopenia risk.
**Table S12:** Population attributable fractions (PAFs) of osteoporosis traits for sarcopenia risk.
**Table S13:** Cross‐sectional associations between osteoporosis traits and sarcopenia prevalence.
**Table S14:** Associations between osteoporosis diagnosis and sarcopenia risk.
**Table S15:** Associations of osteoporosis diagnosis or femoral neck/lumbar spine BMD with confirmed sarcopenia or probable sarcopenia.
**Table S16:** Mendelian randomization analyses for the effects of bone mineral density of lumbar spine on sarcopenia traits.
**Table S17:** Significant proteins mediating the effect of ALM/height^2^ on osteoporosis risk.
**Table S18:** Significant proteins mediating the effect of hand grip strength on osteoporosis risk.
**Table S19:** Significant proteins mediating the effect of usual walking pace on osteoporosis risk.
**Table S20:** Significant metabolites mediating the effect of ALM/height^2^ on osteoporosis risk.
**Table S21:** Significant metabolites mediating the effect of hand grip strength on osteoporosis risk.
**Table S22:** Significant metabolites mediating the effect of usual walking pace on osteoporosis risk.
**Table S23:** Marker gene expressions of different cell types in skeletal muscle.
**Table S24:** Gene expression levels of the significant proteins mediating the effect of ALM/height^2^ on osteoporosis risk.
**Table S25:** Gene expression levels of the significant proteins mediating the effect of usual walking pace on osteoporosis risk.
**Table S26:** Gene expression levels of the significant proteins mediating the effect of hand grip strength on osteoporosis risk.
**Table S27:** Gene expression levels of the significant mediating proteins overlapped across the associations of ALM/height^2^, usual walking pace and hand grip strength with osteoporosis risk.
**Table S28:** The identified regions with significant genetic correlation between osteoporosis risk and sarcopenia risk.
**Table S29:** GWAS results for SNPs within the identified regions that have significant genetic correlation between osteoporosis risk and sarcopenia risk.
**Table S30:** Associations of PRSs with sarcopenia risk and osteoporosis risk.
**Table S31:** Cross‐sectional associations of metabolites with osteoporosis and sarcopenia prevalences.
**Table S32:** Cross‐sectional associations of proteins with osteoporosis and sarcopenia prevalences.
**Table S33:** Tissue enrichment analyses based on GTEx database for significant proteins associated with both osteoporosis and sarcopenia prevalences in consistent effect directions.
**Table S34:** Tissue enrichment analyses based on HPA database for significant proteins associated with both osteoporosis and sarcopenia prevalences in consistent effect directions.
**Table S35:** GO pathway enrichment analyses of significant proteins associated with both osteoporosis and sarcopenia prevalences in consistent effect directions.
**Table S36:** KEGG pathway enrichment analyses of significant proteins associated with both osteoporosis and sarcopenia prevalences in consistent effect directions.
**Table S37:** Transcription factors enrichment profile of significant proteins associated with both osteoporosis and sarcopenia prevalences in consistent effect directions.
**Table S38:** Prediction performance of proteomic and metabolomic signatures for sarcopenia and osteoporosis traits.
**Table S39:** Longitudinal associations of proteomics and metabolomics scores of sarcopenia traits with osteoporosis risk.
**Table S40:** Longitudinal associations of proteomics and metabolomics scores of osteoporosis traits with sarcopenia risk.
**Table S41:** Sample sizes of proteomics and metabolomics scores of sarcopenia and osteoporosis traits.
**Table S42:** Significant metabolites mediating the effect of sarcopenia PRS on sarcopenia prevalence.
**Table S43:** Significant metabolites mediating the effect of sarcopenia PRS on osteoporosis prevalence.
**Table S44:** Significant metabolites mediating the effect of osteoporosis PRS on osteoporosis prevalence.
**Table S45:** Significant metabolites mediating the effect of osteoporosis PRS on sarcopenia prevalence.
**Table S46:** Significant proteins mediating the effect of sarcopenia PRS on sarcopenia prevalence.
**Table S47:** Significant proteins mediating the effect of sarcopenia PRS to osteoporosis prevalence.
**Table S48:** Significant proteins mediating the effect of osteoporosis PRS on osteoporosis prevalence.
**Table S49:** Significant proteins mediating the effect of osteoporosis PRS on sarcopenia prevalence.
**Table S50:** Genes nominally asscociated with either osteoporosis risk or sarcopenia risk in TWAS analyses.
**Table S51:** Genes nominally asscociated with either osteoporosis risk or sarcopenia risk in SMR analyses.
**Table S52:** Numbers of identified genes nominally associated with osteoporosis risk and sarcopenia risk in TWAS (*p* < 0.05) and SMR analysis (SMR *p* < 0.05 and HEIDI *p* > 0.05).
**Table S53:** Significant KEGG pathways for the identified genes nominally associated with both osteoporosis and sarcopenia risks in consistent effect directions.
**Table S54:** Significant GO pathways for the identified genes nominally associated with both osteoporosis and sarcopenia risks in consistent effect directions.
**Table S55:** Enriched transcription factors of the identified genes nominally associated with both osteoporosis and sarcopenia risks in consistent effect directions.
**Table S56:** Cross‐sectional associations of modifiable factors with osteoporosis and sarcopenia prevalences.
**Table S57:** Significant proteins mediating the effects of modifiable factors on osteoporosis and sarcopenia prevalences.
**Table S58:** Significant metabolites mediating the effects of modifiable factors on osteoporosis and sarcopenia prevalences.
**Table S59:** Detailed information of 2923 proteins used in the study.
**Table S60:** Detailed information of 251 metabolites used in the study.
**Table S61:** Summary of the cohorts included in the GWAS meta‐analysis for lumbar spine BMD.

## Data Availability

Summary data involved in the MR analysis can be accessed and downloaded from GWAS Catalog accession (https://www.ebi.ac.uk/gwas/publications/26367794) and MRC‐IEU accession (https://gwas.mrcieu.ac.uk). Researchers registered with the UK Biobank can apply for access to its resources by visiting https://www.ukbiobank.ac.uk/enable‐your‐research/register. All derived datasets and code can be accessed through https://github.com/siqixu/sar‐ost.

## References

[acel70617-bib-0001] Abu‐Amer, Y. 2013. “NF‐κB Signaling and Bone Resorption.” Osteoporosis International 24, no. 9: 2377–2386. 23468073 10.1007/s00198-013-2313-x PMC3884829

[acel70617-bib-0002] Ahmad, S. , A. T. Jan , M. H. Baig , E. J. Lee , and I. Choi . 2017. “Matrix Gla Protein: An Extracellular Matrix Protein Regulates Myostatin Expression in the Muscle Developmental Program.” Life Sciences 172: 55–63. 28012893 10.1016/j.lfs.2016.12.011

[acel70617-bib-0003] Alam, T. I. , T. Kanki , T. Muta , et al. 2003. “Human Mitochondrial DNA Is Packaged With TFAM.” Nucleic Acids Research 31, no. 6: 1640–1645. 12626705 10.1093/nar/gkg251 PMC152855

[acel70617-bib-0004] Anagnostis, P. , M. Florentin , S. Livadas , I. Lambrinoudaki , and D. G. Goulis . 2022. “Bone Health in Patients With Dyslipidemias: An Underestimated Aspect.” International Journal of Molecular Sciences 23, no. 3: 1639. 35163560 10.3390/ijms23031639 PMC8835770

[acel70617-bib-0005] Angelidi, A. M. , K. Stefanakis , S. H. Chou , et al. 2024. “Relative Energy Deficiency in Sport (REDs): Endocrine Manifestations, Pathophysiology and Treatments.” Endocrine Reviews 45, no. 5: 676–708. 38488566 10.1210/endrev/bnae011

[acel70617-bib-0006] Arnson, Y. , Y. Shoenfeld , and H. Amital . 2010. “Effects of Tobacco Smoke on Immunity, Inflammation and Autoimmunity.” Journal of Autoimmunity 34, no. 3: J258–J265. 20042314 10.1016/j.jaut.2009.12.003

[acel70617-bib-0007] Barbeira, A. N. , S. P. Dickinson , R. Bonazzola , et al. 2018. “Exploring the Phenotypic Consequences of Tissue Specific Gene Expression Variation Inferred From GWAS Summary Statistics.” Nature Communications 9, no. 1: 1825. 10.1038/s41467-018-03621-1 PMC5940825 29739930

[acel70617-bib-0008] Barrack, M. T. , J. C. Gibbs , M. J. De Souza , et al. 2014. “Higher Incidence of Bone Stress Injuries With Increasing Female Athlete Triad‐Related Risk Factors: A Prospective Multisite Study of Exercising Girls and Women.” American Journal of Sports Medicine 42, no. 4: 949–958. 24567250 10.1177/0363546513520295

[acel70617-bib-0009] Bi, B. , X. Dong , M. Yan , et al. 2024. “Dyslipidemia Is Associated With Sarcopenia of the Elderly: A Meta‐Analysis.” BMC Geriatrics 24, no. 1: 181. 38395763 10.1186/s12877-024-04761-4 PMC10885450

[acel70617-bib-0010] Bollen, S. E. , J. J. Bass , S. Fujita , D. Wilkinson , M. Hewison , and P. J. Atherton . 2022. “The Vitamin D/Vitamin D Receptor (VDR) Axis in Muscle Atrophy and Sarcopenia.” Cellular Signalling 96: 110355. 35595176 10.1016/j.cellsig.2022.110355

[acel70617-bib-0011] Bowden, J. , G. Davey Smith , P. C. Haycock , and S. Burgess . 2016. “Consistent Estimation in Mendelian Randomization With Some Invalid Instruments Using a Weighted Median Estimator.” Genetic Epidemiology 40, no. 4: 304–314. 27061298 10.1002/gepi.21965 PMC4849733

[acel70617-bib-0012] Brotto, M. , and L. Bonewald . 2015. “Bone and Muscle: Interactions Beyond Mechanical.” Bone 80: 109–114. 26453500 10.1016/j.bone.2015.02.010 PMC4600532

[acel70617-bib-0013] Bulik‐Sullivan, B. , H. K. Finucane , V. Anttila , et al. 2015. “An Atlas of Genetic Correlations Across Human Diseases and Traits.” Nature Genetics 47, no. 11: 1236–1241. 26414676 10.1038/ng.3406 PMC4797329

[acel70617-bib-0014] Burgess, S. , A. Butterworth , and S. G. Thompson . 2013. “Mendelian Randomization Analysis With Multiple Genetic Variants Using Summarized Data.” Genetic Epidemiology 37, no. 7: 658–665. 24114802 10.1002/gepi.21758 PMC4377079

[acel70617-bib-0015] Campbell, K. J. , S. Rocha , and N. D. Perkins . 2004. “Active Repression of Antiapoptotic Gene Expression by RelA(p65) NF‐Kappa B.” Molecular Cell 13, no. 6: 853–865. 15053878 10.1016/s1097-2765(04)00131-5

[acel70617-bib-0016] Carrasco‐Zanini, J. , M. Pietzner , J. Davitte , et al. 2024. “Proteomic Signatures Improve Risk Prediction for Common and Rare Diseases.” Nature Medicine 30, no. 9: 2489–2498. 10.1038/s41591-024-03142-z PMC11405273 39039249

[acel70617-bib-0017] Cartwright, T. , N. D. Perkins , and C. L. Wilson . 2016. “NFKB1: A Suppressor of Inflammation, Ageing and Cancer.” FEBS Journal 283, no. 10: 1812–1822. 26663363 10.1111/febs.13627

[acel70617-bib-0018] Chen, B. , G. Zeng , L. Sun , and C. Jiang . 2024. “When Smoke Meets Gut: Deciphering the Interactions Between Tobacco Smoking and Gut Microbiota in Disease Development.” Science China. Life Sciences 67, no. 5: 854–864. 38265598 10.1007/s11427-023-2446-y

[acel70617-bib-0019] Chen, G. , L. Chen , J. Wen , et al. 2014. “Associations Between Sleep Duration, Daytime Nap Duration, and Osteoporosis Vary by Sex, Menopause, and Sleep Quality.” Journal of Clinical Endocrinology and Metabolism 99, no. 8: 2869–2877. 24848706 10.1210/jc.2013-3629

[acel70617-bib-0020] Chen, S. , X. Xu , H. Gong , et al. 2024. “Global Epidemiological Features and Impact of Osteosarcopenia: A Comprehensive Meta‐Analysis and Systematic Review.” Journal of Cachexia, Sarcopenia and Muscle 15, no. 1: 8–20. 38086772 10.1002/jcsm.13392 PMC10834350

[acel70617-bib-0021] Chen, X. , Y. Ji , R. Liu , et al. 2023. “Mitochondrial Dysfunction: Roles in Skeletal Muscle Atrophy.” Journal of Translational Medicine 21, no. 1: 503. 37495991 10.1186/s12967-023-04369-z PMC10373380

[acel70617-bib-0022] Chen, Y. , F. Zhou , H. Liu , et al. 2021. “SIRT1, a Promising Regulator of Bone Homeostasis.” Life Sciences 269: 119041. 33453243 10.1016/j.lfs.2021.119041

[acel70617-bib-0023] Chen, Y. C. , J. Greenbaum , H. Shen , and H. W. Deng . 2017. “Association Between Gut Microbiota and Bone Health: Potential Mechanisms and Prospective.” Journal of Clinical Endocrinology and Metabolism 102, no. 10: 3635–3646. 28973392 10.1210/jc.2017-00513 PMC5630250

[acel70617-bib-0024] Cheng, L. , and S. Wang . 2023. “Correlation Between Bone Mineral Density and Sarcopenia in US Adults: A Population‐Based Study.” Journal of Orthopaedic Surgery and Research 18, no. 1: 588. 37559054 10.1186/s13018-023-04034-7 PMC10410911

[acel70617-bib-0025] Cheng, Y. , S. Lin , Z. Cao , R. Yu , Y. Fan , and J. Chen . 2025. “The Role of Chronic Low‐Grade Inflammation in the Development of Sarcopenia: Advances in Molecular Mechanisms.” International Immunopharmacology 147: 114056. 39799736 10.1016/j.intimp.2025.114056

[acel70617-bib-0026] Cho, J. , W. Kuswanto , C. Benoist , and D. Mathis . 2019. “T Cell Receptor Specificity Drives Accumulation of a Reparative Population of Regulatory T Cells Within Acutely Injured Skeletal Muscle.” Proceedings of the National Academy of Sciences of the United States of America 116, no. 52: 26727–26733. 31822623 10.1073/pnas.1914848116 PMC6936428

[acel70617-bib-0027] Chung, J. Y. , S. G. Kim , S. H. Kim , and C. H. Park . 2025. “Sarcopenia: How to Determine and Manage.” Knee Surgery & Related Research 37, no. 1: 12. 40098209 10.1186/s43019-025-00265-6 PMC11912661

[acel70617-bib-0028] Clansey, A. C. , M. Hanlon , E. S. Wallace , and M. J. Lake . 2012. “Effects of Fatigue on Running Mechanics Associated With Tibial Stress Fracture Risk.” Medicine and Science in Sports and Exercise 44, no. 10: 1917–1923. 22525776 10.1249/MSS.0b013e318259480d

[acel70617-bib-0029] Clowes, J. A. , B. L. Riggs , and S. Khosla . 2005. “The Role of the Immune System in the Pathophysiology of Osteoporosis.” Immunological Reviews 208: 207–227. 16313351 10.1111/j.0105-2896.2005.00334.x

[acel70617-bib-0030] Connelly, M. A. , J. D. Otvos , I. Shalaurova , M. P. Playford , and N. N. Mehta . 2017. “GlycA, a Novel Biomarker of Systemic Inflammation and Cardiovascular Disease Risk.” Journal of Translational Medicine 15, no. 1: 219. 29078787 10.1186/s12967-017-1321-6 PMC5658936

[acel70617-bib-0031] Cruz‐Jentoft, A. J. , J. P. Baeyens , J. M. Bauer , et al. 2010. “Sarcopenia: European Consensus on Definition and Diagnosis: Report of the European Working Group on Sarcopenia in Older People.” Age and Ageing 39, no. 4: 412–423. 20392703 10.1093/ageing/afq034 PMC2886201

[acel70617-bib-0032] Cruz‐Jentoft, A. J. , G. Bahat , J. Bauer , et al. 2019. “Sarcopenia: Revised European Consensus on Definition and Diagnosis.” Age and Ageing 48, no. 4: 601. 10.1093/ageing/afz046 PMC6593317 31081853

[acel70617-bib-0033] Degens, H. , G. Gayan‐Ramirez , and H. W. van Hees . 2015. “Smoking‐Induced Skeletal Muscle Dysfunction: From Evidence to Mechanisms.” American Journal of Respiratory and Critical Care Medicine 191, no. 6: 620–625. 25581779 10.1164/rccm.201410-1830PP

[acel70617-bib-0034] Dent, E. , J. E. Morley , A. J. Cruz‐Jentoft , et al. 2018. “International Clinical Practice Guidelines for Sarcopenia (ICFSR): Screening, Diagnosis and Management.” Journal of Nutrition, Health & Aging 22, no. 10: 1148–1161. 10.1007/s12603-018-1139-9 PMC12280515 30498820

[acel70617-bib-0035] DiGirolamo, D. J. , D. P. Kiel , and K. A. Esser . 2013. “Bone and Skeletal Muscle: Neighbors With Close Ties.” Journal of Bone and Mineral Research 28, no. 7: 1509–1518. 23630111 10.1002/jbmr.1969 PMC4892934

[acel70617-bib-0036] Dodds, R. M. , A. Granic , S. M. Robinson , and A. A. Sayer . 2020. “Sarcopenia, Long‐Term Conditions, and Multimorbidity: Findings From UK Biobank Participants.” Journal of Cachexia, Sarcopenia and Muscle 11, no. 1: 62–68. 31886632 10.1002/jcsm.12503 PMC7015236

[acel70617-bib-0037] Eriksen, E. F. 2012. “Treatment of Osteopenia.” Reviews in Endocrine & Metabolic Disorders 13, no. 3: 209–223. 21710179 10.1007/s11154-011-9187-z PMC3411311

[acel70617-bib-0038] Farr, J. N. , D. G. Fraser , H. Wang , et al. 2016. “Identification of Senescent Cells in the Bone Microenvironment.” Journal of Bone and Mineral Research 31, no. 11: 1920–1929. 27341653 10.1002/jbmr.2892 PMC5289710

[acel70617-bib-0039] Ferretti, J. L. , G. R. Cointry , R. F. Capozza , and H. M. Frost . 2003. “Bone Mass, Bone Strength, Muscle‐Bone Interactions, Osteopenias and Osteoporoses.” Mechanisms of Ageing and Development 124, no. 3: 269–279. 12663124 10.1016/s0047-6374(02)00194-x

[acel70617-bib-0040] Fricker, M. , B. J. Goggins , S. Mateer , et al. 2018. “Chronic Cigarette Smoke Exposure Induces Systemic Hypoxia That Drives Intestinal Dysfunction.” JCI Insight 3, no. 3: e94040. 29415878 10.1172/jci.insight.94040 PMC5821186

[acel70617-bib-0041] Gao, T. , Z. Wang , Y. Dong , et al. 2019. “Role of Melatonin in Sleep Deprivation‐Induced Intestinal Barrier Dysfunction in Mice.” Journal of Pineal Research 67, no. 1: e12574. 30929267 10.1111/jpi.12574

[acel70617-bib-0042] Gielen, E. , J. Dupont , M. Dejaeger , and M. R. Laurent . 2023. “Sarcopenia, Osteoporosis and Frailty.” Metabolism, Clinical and Experimental 145: 155638. 37348597 10.1016/j.metabol.2023.155638

[acel70617-bib-0043] Goeminne, L. J. E. , A. Vladimirova , A. Eames , et al. 2025. “Plasma Protein‐Based Organ‐Specific Aging and Mortality Models Unveil Diseases as Accelerated Aging of Organismal Systems.” Cell Metabolism 37, no. 1: 205–222.e6. 39488213 10.1016/j.cmet.2024.10.005

[acel70617-bib-0044] GTEx Consortium . 2013. “The Genotype‐Tissue Expression (GTEx) Project.” Nature Genetics 45, no. 6: 580–585. 23715323 10.1038/ng.2653 PMC4010069

[acel70617-bib-0045] Guadalupe‐Grau, A. , T. Fuentes , B. Guerra , and J. A. Calbet . 2009. “Exercise and Bone Mass in Adults.” Sports Medicine 39, no. 6: 439–468. 19453205 10.2165/00007256-200939060-00002

[acel70617-bib-0046] Hart, N. H. , S. Nimphius , T. Rantalainen , A. Ireland , A. Siafarikas , and R. U. Newton . 2017. “Mechanical Basis of Bone Strength: Influence of Bone Material, Bone Structure and Muscle Action.” Journal of Musculoskeletal & Neuronal Interactions 17, no. 3: 114–139. 28860414 PMC5601257

[acel70617-bib-0047] Hemani, G. , K. Tilling , and G. Davey Smith . 2017. “Orienting the Causal Relationship Between Imprecisely Measured Traits Using GWAS Summary Data.” PLoS Genetics 13, no. 11: e1007081. 29149188 10.1371/journal.pgen.1007081 PMC5711033

[acel70617-bib-0048] Herman, B. C. , L. Cardoso , R. J. Majeska , K. J. Jepsen , and M. B. Schaffler . 2010. “Activation of Bone Remodeling After Fatigue: Differential Response to Linear Microcracks and Diffuse Damage.” Bone 47, no. 4: 766–772. 20633708 10.1016/j.bone.2010.07.006 PMC2939191

[acel70617-bib-0049] Hernlund, E. , A. Svedbom , M. Ivergård , et al. 2013. “Osteoporosis in the European Union: Medical Management, Epidemiology and Economic Burden. A Report Prepared in Collaboration With the International Osteoporosis Foundation (IOF) and the European Federation of Pharmaceutical Industry Associations (EFPIA).” Archives of Osteoporosis 8, no. 1: 136. 24113837 10.1007/s11657-013-0136-1 PMC3880487

[acel70617-bib-0050] Ho‐Pham, L. T. , U. D. Nguyen , and T. V. Nguyen . 2014. “Association Between Lean Mass, Fat Mass, and Bone Mineral Density: A Meta‐Analysis.” Journal of Clinical Endocrinology and Metabolism 99, no. 1: 30–38. 24384013 10.1210/jc.2014-v99i12-30A

[acel70617-bib-0051] Huang, Y. H. , W. C. Chiu , Y. P. Hsu , Y. L. Lo , and Y. H. Wang . 2020. “Effects of Omega‐3 Fatty Acids on Muscle Mass, Muscle Strength and Muscle Performance Among the Elderly: A Meta‐Analysis.” Nutrients 12, no. 12: 3739. 33291698 10.3390/nu12123739 PMC7761957

[acel70617-bib-0052] Jiang, L. , Z. Zheng , H. Fang , and J. Yang . 2021. “A Generalized Linear Mixed Model Association Tool for Biobank‐Scale Data.” Nature Genetics 53, no. 11: 1616–1621. 34737426 10.1038/s41588-021-00954-4

[acel70617-bib-0053] Johann, K. , M. Kleinert , and S. Klaus . 2021. “The Role of GDF15 as a Myomitokine.” Cells 10, no. 11: 2990. 34831213 10.3390/cells10112990 PMC8616340

[acel70617-bib-0054] Julien, M. , S. Khoshniat , A. Lacreusette , et al. 2009. “Phosphate‐Dependent Regulation of MGP in Osteoblasts: Role of ERK1/2 and Fra‐1.” Journal of Bone and Mineral Research 24, no. 11: 1856–1868. 19419315 10.1359/jbmr.090508

[acel70617-bib-0055] Kaetzel, C. S. 2005. “The Polymeric Immunoglobulin Receptor: Bridging Innate and Adaptive Immune Responses at Mucosal Surfaces.” Immunological Reviews 206: 83–99. 16048543 10.1111/j.0105-2896.2005.00278.x

[acel70617-bib-0056] Kedlian, V. R. , Y. Wang , T. Liu , et al. 2024. “Human Skeletal Muscle Aging Atlas.” Nature Aging 4, no. 5: 727–744. 38622407 10.1038/s43587-024-00613-3 PMC11108788

[acel70617-bib-0057] Khan, N. A. , G. Lawyer , S. McDonough , et al. 2020. “Systemic Biomarkers of Inflammation, Oxidative Stress and Tissue Injury and Repair Among Waterpipe, Cigarette and Dual Tobacco Smokers.” Tobacco Control 29, no. S2: s102–s109. 31494573 10.1136/tobaccocontrol-2019-054958 PMC7050420

[acel70617-bib-0058] Kim, N. , H. R. Choi , S. W. Kim , B. S. Kim , C. W. Won , and S. Y. Kim . 2014. “Association Between Bone Mineral Density and Sleep Duration in the Korean Elderly Population.” Korean Journal of Family Medicine 35, no. 2: 90–97. 24724004 10.4082/kjfm.2014.35.2.90 PMC3978190

[acel70617-bib-0059] Kuriyama, N. , M. Inaba , E. Ozaki , et al. 2017. “Association Between Loss of Bone Mass due to Short Sleep and Leptin‐Sympathetic Nervous System Activity.” Archives of Gerontology and Geriatrics 70: 201–208. 28214401 10.1016/j.archger.2017.02.005

[acel70617-bib-0060] Lee, D. , and A. L. Goldberg . 2013. “SIRT1 Protein, by Blocking the Activities of Transcription Factors FoxO1 and FoxO3, Inhibits Muscle Atrophy and Promotes Muscle Growth.” Journal of Biological Chemistry 288, no. 42: 30515–30526. 24003218 10.1074/jbc.M113.489716 PMC3798522

[acel70617-bib-0061] Lee, D. Y. , and S. Shin . 2021. “Association of Sarcopenia With Osteopenia and Osteoporosis in Community‐Dwelling Older Korean Adults: A Cross‐Sectional Study.” Journal of Clinical Medicine 11, no. 1: 129. 35011870 10.3390/jcm11010129 PMC8745168

[acel70617-bib-0062] Li, C. W. , K. Yu , N. Shyh‐Chang , et al. 2022. “Pathogenesis of Sarcopenia and the Relationship With Fat Mass: Descriptive Review.” Journal of Cachexia, Sarcopenia and Muscle 13, no. 2: 781–794. 35106971 10.1002/jcsm.12901 PMC8977978

[acel70617-bib-0063] Li, X. , J. He , and Q. Sun . 2023. “Sleep Duration and Sarcopenia: An Updated Systematic Review and Meta‐Analysis.” Journal of the American Medical Directors Association 24, no. 8: 1193–1206.e5. 37295459 10.1016/j.jamda.2023.04.032

[acel70617-bib-0064] Lipina, C. , and H. S. Hundal . 2017. “Lipid Modulation of Skeletal Muscle Mass and Function.” Journal of Cachexia, Sarcopenia and Muscle 8, no. 2: 190–201. 27897400 10.1002/jcsm.12144 PMC5377414

[acel70617-bib-0065] Liu, D. , S. Wang , S. Liu , Q. Wang , X. Che , and G. Wu . 2024. “Frontiers in Sarcopenia: Advancements in Diagnostics, Molecular Mechanisms, and Therapeutic Strategies.” Molecular Aspects of Medicine 97: 101270. 38583268 10.1016/j.mam.2024.101270

[acel70617-bib-0066] Liu, J. , S. Wang , Y. Shen , H. Shi , and L. Han . 2024. “Lipid Metabolites and Sarcopenia‐Related Traits: A Mendelian Randomization Study.” Diabetology & Metabolic Syndrome 16, no. 1: 231. 39285470 10.1186/s13098-024-01465-y PMC11406728

[acel70617-bib-0067] Liu, T. , L. Zhang , D. Joo , and S.‐C. Sun . 2017. “NF‐κB Signaling in Inflammation.” Signal Transduction and Targeted Therapy 2, no. 1: 1–9. 10.1038/sigtrans.2017.23 PMC5661633 29158945

[acel70617-bib-0068] Liu, X. , G. Yang , Y. Li , W. Xiao , B. Lu , and Y. Wang . 2025. “Causal Relationship Between Blood Metabolites and Osteoporosis: A Two‐Sample Mendelian Randomization and Genetic Correlation Analysis.” Bioengineering 12, no. 5: 435. 40428054 10.3390/bioengineering12050435 PMC12109198

[acel70617-bib-0069] Liu, Z. , X. Chen , Z. Ruan , et al. 2025. “Genetic Analysis of Comorbidities Between Osteoarthritis, Sarcopenia, and Osteoporosis.” Experimental Gerontology 206: 112788. 40389141 10.1016/j.exger.2025.112788

[acel70617-bib-0070] Livshits, G. , and A. Kalinkovich . 2022. “Targeting Chronic Inflammation as a Potential Adjuvant Therapy for Osteoporosis.” Life Sciences 306: 120847. 35908619 10.1016/j.lfs.2022.120847

[acel70617-bib-0071] Lourida, I. , E. Hannon , T. J. Littlejohns , et al. 2019. “Association of Lifestyle and Genetic Risk With Incidence of Dementia.” JAMA 322, no. 5: 430–437. 31302669 10.1001/jama.2019.9879 PMC6628594

[acel70617-bib-0072] McGrath, R. P. , W. J. Kraemer , B. M. Vincent , O. T. Hall , and M. D. Peterson . 2017. “Muscle Strength Is Protective Against Osteoporosis in an Ethnically Diverse Sample of Adults.” Journal of Strength and Conditioning Research 31, no. 9: 2586–2589. 28658086 10.1519/JSC.0000000000002080

[acel70617-bib-0073] Milgrom, C. , D. R. Radeva‐Petrova , A. Finestone , et al. 2007. “The Effect of Muscle Fatigue on In Vivo Tibial Strains.” Journal of Biomechanics 40, no. 4: 845–850. 16682046 10.1016/j.jbiomech.2006.03.006

[acel70617-bib-0074] Montserrat‐de la Paz, S. , M. Del Carmen Naranjo , S. Lopez , et al. 2023. “Immediate‐Release Niacin and a Monounsaturated Fatty Acid‐Rich Meal on Postprandial Inflammation and Monocyte Characteristics in Men With Metabolic Syndrome.” Clinical Nutrition 42, no. 11: 2138–2150. 37774650 10.1016/j.clnu.2023.08.017

[acel70617-bib-0075] Moon, S. S. 2014. “Relationship of Lean Body Mass With Bone Mass and Bone Mineral Density in the General Korean Population.” Endocrine 47, no. 1: 234–243. 24415174 10.1007/s12020-013-0160-3

[acel70617-bib-0076] Morgese, M. G. , S. Schiavone , M. Bove , et al. 2021. “N‐3 PUFA Prevent Oxidative Stress in a Rat Model of Beta‐Amyloid‐Induced Toxicity.” Pharmaceuticals 14, no. 4: 339. 33917814 10.3390/ph14040339 PMC8068120

[acel70617-bib-0077] Mullington, J. M. , N. S. Simpson , H. K. Meier‐Ewert , and M. Haack . 2010. “Sleep Loss and Inflammation.” Best Practice & Research—Clinical Endocrinology & Metabolism 24, no. 5: 775–784. 21112025 10.1016/j.beem.2010.08.014 PMC3548567

[acel70617-bib-0078] Nakamichi, Y. , N. Udagawa , K. Horibe , et al. 2017. “VDR in Osteoblast‐Lineage Cells Primarily Mediates Vitamin D Treatment‐Induced Increase in Bone Mass by Suppressing Bone Resorption.” Journal of Bone and Mineral Research 32, no. 6: 1297–1308. 28177161 10.1002/jbmr.3096

[acel70617-bib-0079] Nattiv, A. , A. B. Loucks , M. M. Manore , C. F. Sanborn , J. Sundgot‐Borgen , and M. P. Warren . 2007. “American College of Sports Medicine Position Stand. The Female Athlete Triad.” Medicine and Science in Sports and Exercise 39, no. 10: 1867–1882. 17909417 10.1249/mss.0b013e318149f111

[acel70617-bib-0080] Neefjes, J. , M. L. Jongsma , P. Paul , and O. Bakke . 2011. “Towards a Systems Understanding of MHC Class I and MHC Class II Antigen Presentation.” Nature Reviews Immunology 11, no. 12: 823–836. 10.1038/nri3084 22076556

[acel70617-bib-0081] Nelke, C. , R. Dziewas , J. Minnerup , S. G. Meuth , and T. Ruck . 2019. “Skeletal Muscle as Potential Central Link Between Sarcopenia and Immune Senescence.” eBioMedicine 49: 381–388. 31662290 10.1016/j.ebiom.2019.10.034 PMC6945275

[acel70617-bib-0082] Nguyen, A. , P. Lee , E. K. Rodriguez , K. Chahal , B. R. Freedman , and A. Nazarian . 2025. “Addressing the Growing Burden of Musculoskeletal Diseases in the Ageing US Population: Challenges and Innovations.” Lancet Healthy Longevity 6, no. 5: 100707. 40381641 10.1016/j.lanhl.2025.100707

[acel70617-bib-0083] Nishikawa, H. , S. Nakamura , T. Miyazaki , et al. 2021. “Inflammatory Bowel Disease and Sarcopenia: Its Mechanism and Clinical Importance.” Journal of Clinical Medicine 10, no. 18: 4214. 34575326 10.3390/jcm10184214 PMC8470813

[acel70617-bib-0084] Novotny, S. A. , G. L. Warren , and M. W. Hamrick . 2015. “Aging and the Muscle‐Bone Relationship.” Physiology (Bethesda) 30, no. 1: 8–16. 25559151 10.1152/physiol.00033.2014 PMC4285576

[acel70617-bib-0085] Nowak, A. , and M. Ogurkowska . 2024. “Bone Health and Physical Activity—The Complex Mechanism.” Aging and Disease 16, no. 6: 3400–3420. 39751867 10.14336/AD.2024.1316 PMC12539549

[acel70617-bib-0086] Ochs‐Balcom, H. M. , K. M. Hovey , C. Andrews , et al. 2020. “Short Sleep Is Associated With Low Bone Mineral Density and Osteoporosis in the Women's Health Initiative.” Journal of Bone and Mineral Research 35, no. 2: 261–268. 31692127 10.1002/jbmr.3879 PMC8223077

[acel70617-bib-0087] Oh, H. S. , J. Rutledge , D. Nachun , et al. 2023. “Organ Aging Signatures in the Plasma Proteome Track Health and Disease.” Nature 624, no. 7990: 164–172. 38057571 10.1038/s41586-023-06802-1 PMC10700136

[acel70617-bib-0088] Ohlsson, C. , and K. Sjögren . 2015. “Effects of the Gut Microbiota on Bone Mass.” Trends in Endocrinology and Metabolism: TEM 26, no. 2: 69–74. 25497348 10.1016/j.tem.2014.11.004

[acel70617-bib-0089] Orr, W. C. , R. Fass , S. S. Sundaram , and A. O. Scheimann . 2020. “The Effect of Sleep on Gastrointestinal Functioning in Common Digestive Diseases.” Lancet Gastroenterology & Hepatology 5, no. 6: 616–624. 32416862 10.1016/S2468-1253(19)30412-1

[acel70617-bib-0090] Pacifici, R. 2016. “T Cells, Osteoblasts, and Osteocytes: Interacting Lineages Key for the Bone Anabolic and Catabolic Activities of Parathyroid Hormone.” Annals of the New York Academy of Sciences 1364, no. 1: 11–24. 26662934 10.1111/nyas.12969 PMC4803611

[acel70617-bib-0091] Pan, Y. , and J. Xu . 2022. “Association Between Muscle Mass, Bone Mineral Density and Osteoporosis in Type 2 Diabetes.” Journal of Diabetes Investigation 13, no. 2: 351–358. 34342165 10.1111/jdi.13642 PMC8847116

[acel70617-bib-0092] Patel, S. R. , X. Zhu , A. Storfer‐Isser , et al. 2009. “Sleep Duration and Biomarkers of Inflammation.” Sleep 32, no. 2: 200–204. 19238807 10.1093/sleep/32.2.200 PMC2635584

[acel70617-bib-0093] Pedersen, B. K. 2009. “The Diseasome of Physical Inactivity—And the Role of Myokines in Muscle—Fat Cross Talk.” Journal of Physiology 587, no. pt. 23: 5559–5568. 19752112 10.1113/jphysiol.2009.179515 PMC2805368

[acel70617-bib-0094] Petermann‐Rocha, F. , V. Balntzi , S. R. Gray , et al. 2022. “Global Prevalence of Sarcopenia and Severe Sarcopenia: A Systematic Review and Meta‐Analysis.” Journal of Cachexia, Sarcopenia and Muscle 13, no. 1: 86–99. 34816624 10.1002/jcsm.12783 PMC8818604

[acel70617-bib-0095] Piovezan, R. D. , J. Abucham , R. V. Dos Santos , M. T. Mello , S. Tufik , and D. Poyares . 2015. “The Impact of Sleep on Age‐Related Sarcopenia: Possible Connections and Clinical Implications.” Ageing Research Reviews 23, no. pt. B: 210–220. 26216211 10.1016/j.arr.2015.07.003

[acel70617-bib-0096] Pober, J. S. , and W. C. Sessa . 2007. “Evolving Functions of Endothelial Cells in Inflammation.” Nature Reviews Immunology 7, no. 10: 803–815. 10.1038/nri2171 17893694

[acel70617-bib-0097] Rached, M. T. , A. Kode , L. Xu , et al. 2010. “FoxO1 Is a Positive Regulator of Bone Formation by Favoring Protein Synthesis and Resistance to Oxidative Stress in Osteoblasts.” Cell Metabolism 11, no. 2: 147–160. 20142102 10.1016/j.cmet.2010.01.001 PMC2820405

[acel70617-bib-0098] Rachner, T. D. , S. Khosla , and L. C. Hofbauer . 2011. “Osteoporosis: Now and the Future.” Lancet 377, no. 9773: 1276–1287. 21450337 10.1016/S0140-6736(10)62349-5 PMC3555696

[acel70617-bib-0099] Rikkonen, T. , J. Sirola , K. Salovaara , et al. 2012. “Muscle Strength and Body Composition Are Clinical Indicators of Osteoporosis.” Calcified Tissue International 91, no. 2: 131–138. 22733383 10.1007/s00223-012-9618-1

[acel70617-bib-0100] Rozengurt, E. , and J. H. Walsh . 2001. “Gastrin, CCK, Signaling, and Cancer.” Annual Review of Physiology 63: 49–76. 10.1146/annurev.physiol.63.1.49 11181948

[acel70617-bib-0101] Rozner, R. , J. Vernikov , S. Griess‐Fishheimer , et al. 2020. “The Role of Omega‐3 Polyunsaturated Fatty Acids From Different Sources in Bone Development.” Nutrients 12, no. 11: 3494. 33202985 10.3390/nu12113494 PMC7697266

[acel70617-bib-0102] Rubio‐Arias, J. , R. Rodríguez‐Fernández , L. Andreu , L. M. Martínez‐Aranda , A. Martínez‐Rodriguez , and D. J. Ramos‐Campo . 2019. “Effect of Sleep Quality on the Prevalence of Sarcopenia in Older Adults: A Systematic Review With Meta‐Analysis.” Journal of Clinical Medicine 8, no. 12: 2156. 31817603 10.3390/jcm8122156 PMC6947616

[acel70617-bib-0103] Saint Martin, M. , P. Labeix , M. Garet , et al. 2016. “Does Subjective Sleep Affect Bone Mineral Density in Older People With Minimal Health Disorders? The PROOF Cohort.” Journal of Clinical Sleep Medicine 12, no. 11: 1461–1469. 27655463 10.5664/jcsm.6266 PMC5078700

[acel70617-bib-0104] Salari, N. , N. Darvishi , Y. Bartina , et al. 2021. “Global Prevalence of Osteoporosis Among the World Older Adults: A Comprehensive Systematic Review and Meta‐Analysis.” Journal of Orthopaedic Surgery and Research 16, no. 1: 669. 34774085 10.1186/s13018-021-02821-8 PMC8590304

[acel70617-bib-0105] Schrauwen, P. 2007. “High‐Fat Diet, Muscular Lipotoxicity and Insulin Resistance.” Proceedings of the Nutrition Society 66, no. 1: 33–41. 17343770 10.1017/S0029665107005277

[acel70617-bib-0106] Schreiber, T. H. , D. Wolf , M. Bodero , L. Gonzalez , and E. R. Podack . 2012. “T Cell Costimulation by TNFR Superfamily (TNFRSF)4 and TNFRSF25 in the Context of Vaccination.” Journal of Immunology 189, no. 7: 3311–3318. 10.4049/jimmunol.1200597 PMC3449097 22956587

[acel70617-bib-0107] Shi, Y. , Y. Wang , Q. Li , et al. 2018. “Immunoregulatory Mechanisms of Mesenchymal Stem and Stromal Cells in Inflammatory Diseases.” Nature Reviews Nephrology 14, no. 8: 493–507. 29895977 10.1038/s41581-018-0023-5

[acel70617-bib-0108] Sin, T. K. , B. Y. Yung , and P. M. Siu . 2015. “Modulation of SIRT1‐Foxo1 Signaling Axis by Resveratrol: Implications in Skeletal Muscle Aging and Insulin Resistance.” Cellular Physiology and Biochemistry 35, no. 2: 541–552. 25612477 10.1159/000369718

[acel70617-bib-0109] Smith, L. L. 2000. “Cytokine Hypothesis of Overtraining: A Physiological Adaptation to Excessive Stress?” Medicine and Science in Sports and Exercise 32, no. 2: 317–331. 10694113 10.1097/00005768-200002000-00011

[acel70617-bib-0110] Srivastava, R. K. , H. Y. Dar , and P. K. Mishra . 2018. “Immunoporosis: Immunology of Osteoporosis‐Role of T Cells.” Frontiers in Immunology 9: 657. 29675022 10.3389/fimmu.2018.00657 PMC5895643

[acel70617-bib-0111] Sudlow, C. , J. Gallacher , N. Allen , et al. 2015. “UK Biobank: An Open Access Resource for Identifying the Causes of a Wide Range of Complex Diseases of Middle and Old Age.” PLoS Medicine 12, no. 3: e1001779. 25826379 10.1371/journal.pmed.1001779 PMC4380465

[acel70617-bib-0112] Tagliaferri, C. , Y. Wittrant , M. J. Davicco , S. Walrand , and V. Coxam . 2015. “Muscle and Bone, Two Interconnected Tissues.” Ageing Research Reviews 21: 55–70. 25804855 10.1016/j.arr.2015.03.002

[acel70617-bib-0113] Taupin, D. , and D. K. Podolsky . 2003. “Trefoil Factors: Initiators of Mucosal Healing.” Nature Reviews Molecular Cell Biology 4, no. 9: 721–732. 14506475 10.1038/nrm1203

[acel70617-bib-0114] Teng, Z. , Y. Zhu , Y. Teng , et al. 2021. “The Analysis of Osteosarcopenia as a Risk Factor for Fractures, Mortality, and Falls.” Osteoporosis International 32, no. 11: 2173–2183. 33877382 10.1007/s00198-021-05963-x

[acel70617-bib-0115] Thunø, M. , B. Macho , and J. Eugen‐Olsen . 2009. “suPAR: The Molecular Crystal Ball.” Disease Markers 27, no. 3: 157–172. 19893210 10.3233/DMA-2009-0657 PMC3835059

[acel70617-bib-0116] Ticinesi, A. , A. Nouvenne , N. Cerundolo , et al. 2019. “Gut Microbiota, Muscle Mass and Function in Aging: A Focus on Physical Frailty and Sarcopenia.” Nutrients 11, no. 7: 1633. 31319564 10.3390/nu11071633 PMC6683074

[acel70617-bib-0117] Tidball, J. G. , and S. A. Villalta . 2010. “Regulatory Interactions Between Muscle and the Immune System During Muscle Regeneration.” American Journal of Physiology—Regulatory, Integrative and Comparative Physiology 298, no. 5: R1173–R1187. 20219869 10.1152/ajpregu.00735.2009 PMC2867520

[acel70617-bib-0118] Travis, C. , P. S. Srivastava , T. J. Hawke , and E. Kalaitzoglou . 2022. “Diabetic Bone Disease and Diabetic Myopathy: Manifestations of the Impaired Muscle‐Bone Unit in Type 1 Diabetes.” Journal of Diabetes Research 2022: 2650342. 35601019 10.1155/2022/2650342 PMC9119786

[acel70617-bib-0119] Uhlén, M. , L. Fagerberg , B. M. Hallström , et al. 2015. “Tissue‐Based Map of the Human Proteome.” Science 347, no. 6220: 1260419. 25613900 10.1126/science.1260419

[acel70617-bib-0120] van Dijk, S. J. , E. J. Feskens , M. B. Bos , et al. 2009. “A Saturated Fatty Acid‐Rich Diet Induces an Obesity‐Linked Proinflammatory Gene Expression Profile in Adipose Tissue of Subjects at Risk of Metabolic Syndrome.” American Journal of Clinical Nutrition 90, no. 6: 1656–1664. 19828712 10.3945/ajcn.2009.27792

[acel70617-bib-0121] Villard, J. , M. Peretti , K. Masternak , et al. 2000. “A Functionally Essential Domain of RFX5 Mediates Activation of Major Histocompatibility Complex Class II Promoters by Promoting Cooperative Binding Between RFX and NF‐Y.” Molecular and Cellular Biology 20, no. 10: 3364–3376. 10779326 10.1128/mcb.20.10.3364-3376.2000 PMC85629

[acel70617-bib-0122] Wang, T. , D. Zhou , and Z. Hong . 2024. “Adipose Tissue in Older Individuals: A Contributing Factor to Sarcopenia.” Metabolism 160: 155998. 39128607 10.1016/j.metabol.2024.155998

[acel70617-bib-0123] Weber, D. R. , and G. Schwartz . 2016. “Epidemiology of Skeletal Health in Type 1 Diabetes.” Current Osteoporosis Reports 14, no. 6: 327–336. 27744554 10.1007/s11914-016-0333-0 PMC5130154

[acel70617-bib-0124] Werme, J. , S. van der Sluis , D. Posthuma , and C. A. de Leeuw . 2022. “An Integrated Framework for Local Genetic Correlation Analysis.” Nature Genetics 54, no. 3: 274–282. 35288712 10.1038/s41588-022-01017-y

[acel70617-bib-0125] Xiao, H. , W. Li , Y. Qin , et al. 2024. “Crosstalk Between Lipid Metabolism and Bone Homeostasis: Exploring Intricate Signaling Relationships.” Research 7: 0447. 39165638 10.34133/research.0447 PMC11334918

[acel70617-bib-0126] Yoon, V. , N. M. Maalouf , and K. Sakhaee . 2012. “The Effects of Smoking on Bone Metabolism.” Osteoporosis International 23, no. 8: 2081–2092. 22349964 10.1007/s00198-012-1940-y

[acel70617-bib-0127] Yu, R. , J. Leung , and J. Woo . 2014. “Incremental Predictive Value of Sarcopenia for Incident Fracture in an Elderly Chinese Cohort: Results From the Osteoporotic Fractures in Men (MrOs) Study.” Journal of the American Medical Directors Association 15, no. 8: 551–558. 24703927 10.1016/j.jamda.2014.02.005

[acel70617-bib-0128] Yu, X. , S. Sun , S. Zhang , et al. 2022. “A Pooled Analysis of the Association Between Sarcopenia and Osteoporosis.” Medicine (Baltimore) 101, no. 46: e31692. 36401390 10.1097/MD.0000000000031692 PMC9678526

[acel70617-bib-0129] Zhang, Q. , J. Zhou , Q. Wang , et al. 2020. “Association Between Bone Mineral Density and Lipid Profile in Chinese Women.” Clinical Interventions in Aging 15: 1649–1664. 32982199 10.2147/CIA.S266722 PMC7501971

[acel70617-bib-0130] Zhang, W. , R. Gao , X. Rong , et al. 2022. “Immunoporosis: Role of Immune System in the Pathophysiology of Different Types of Osteoporosis.” Frontiers in Endocrinology (Lausanne) 13: 965258. 10.3389/fendo.2022.965258 PMC9487180 36147571

[acel70617-bib-0131] Zheng, C. X. , B. D. Sui , X. Y. Qiu , C. H. Hu , and Y. Jin . 2020. “Mitochondrial Regulation of Stem Cells in Bone Homeostasis.” Trends in Molecular Medicine 26, no. 1: 89–104. 31126872 10.1016/j.molmed.2019.04.008

[acel70617-bib-0132] Zheng, H. F. , V. Forgetta , Y. H. Hsu , et al. 2015. “Whole‐Genome Sequencing Identifies EN1 as a Determinant of Bone Density and Fracture.” Nature 526, no. 7571: 112–117. 26367794 10.1038/nature14878 PMC4755714

[acel70617-bib-0133] Zheng, L. , N. You , X. Huang , et al. 2019. “COMMD7 Regulates NF‐κB Signaling Pathway in Hepatocellular Carcinoma Stem‐Like Cells.” Molecular Therapy—Oncolytics 12: 112–123. 30719501 10.1016/j.omto.2018.12.006 PMC6350112

[acel70617-bib-0134] Zhu, L. , F. Hua , W. Ding , K. Ding , Y. Zhang , and C. Xu . 2020. “The Correlation Between the Th17/Treg Cell Balance and Bone Health.” Immunity & Ageing 17: 30. 33072163 10.1186/s12979-020-00202-z PMC7557094

[acel70617-bib-0135] Zhu, Z. , F. Zhang , H. Hu , et al. 2016. “Integration of Summary Data From GWAS and eQTL Studies Predicts Complex Trait Gene Targets.” Nature Genetics 48, no. 5: 481–487. 27019110 10.1038/ng.3538

