# Supplementary material for: Bidirectional Relationship and Shared Mechanisms Between Sarcopenia and Osteoporosis: An Observational Study Integrating Genomic, Proteomic, and Metabolomic Data
Source: Aging Cell. 2026 Jun 30;25(7):e70617. doi: 10.1111/acel.70617 (PMC13318683; doi:10.1111/acel.70617)
Supplement: Supplementary file 1 — Figure S1: Exposure–response relationships between osteoporosis traits and sarcopenia. Exposure–response curves depicting odd ratios and 95% confidence intervals for sarcopenia risk against osteoporosis traits under restricted cubic spline regressions. Histograms display sample distributions of each trait. BMD, bone mineral density. Figure S2: Marker gene expressions of different cell types in skeletal muscle. Average expression represents the average of standardized gene expression levels within each cell type. Percent expression denotes the percentage of cells expressing the given gene within each cell type. Figure S3: Scatter plots of SNP effect sizes for sarcopenia risk (x‐axis) and osteoporosis risk (y‐axis) in genomic regions with significant local genetic correlation. Red and blue dots denote SNPs significantly associated with both traits (p < 0.05) in consistent and opposite directions, respectively. Gray dots represent other SNPs in these regions. The slope of dashed line indicates the estimated local genetic correlation (r). r and p denote the local genetic correlation estimate and corresponding p value, respectively. Figure S4: Protein–protein interaction (PPI) network for proteins associated with both osteoporosis and sarcopenia in consistent effect directions. The PPI network has a clustering coefficient of 0.318, containing 496 nodes and 353 edges (expected number of edges: 67, enrichment p value < 1 × 10^−16^). Figure S5: Transcription factors enrichment profile of proteins associated with both osteoporosis risk and sarcopenia risk. Figure S6: Associations of proteomic and metabolomic signatures for sarcopenia traits with osteoporosis risk. (A, B) Survival curves for osteoporosis onset stratified by (A) proteomic and (B) metabolomic scores for sarcopenia traits. (C) Forest plot of hazard ratios and 95% confidence intervals for the associations of proteomic and metabolomic scores of sarcopenia traits with osteoporosis risk. Figure S7: Exposure–response r [file ACEL-25-e70617-s001.docx]

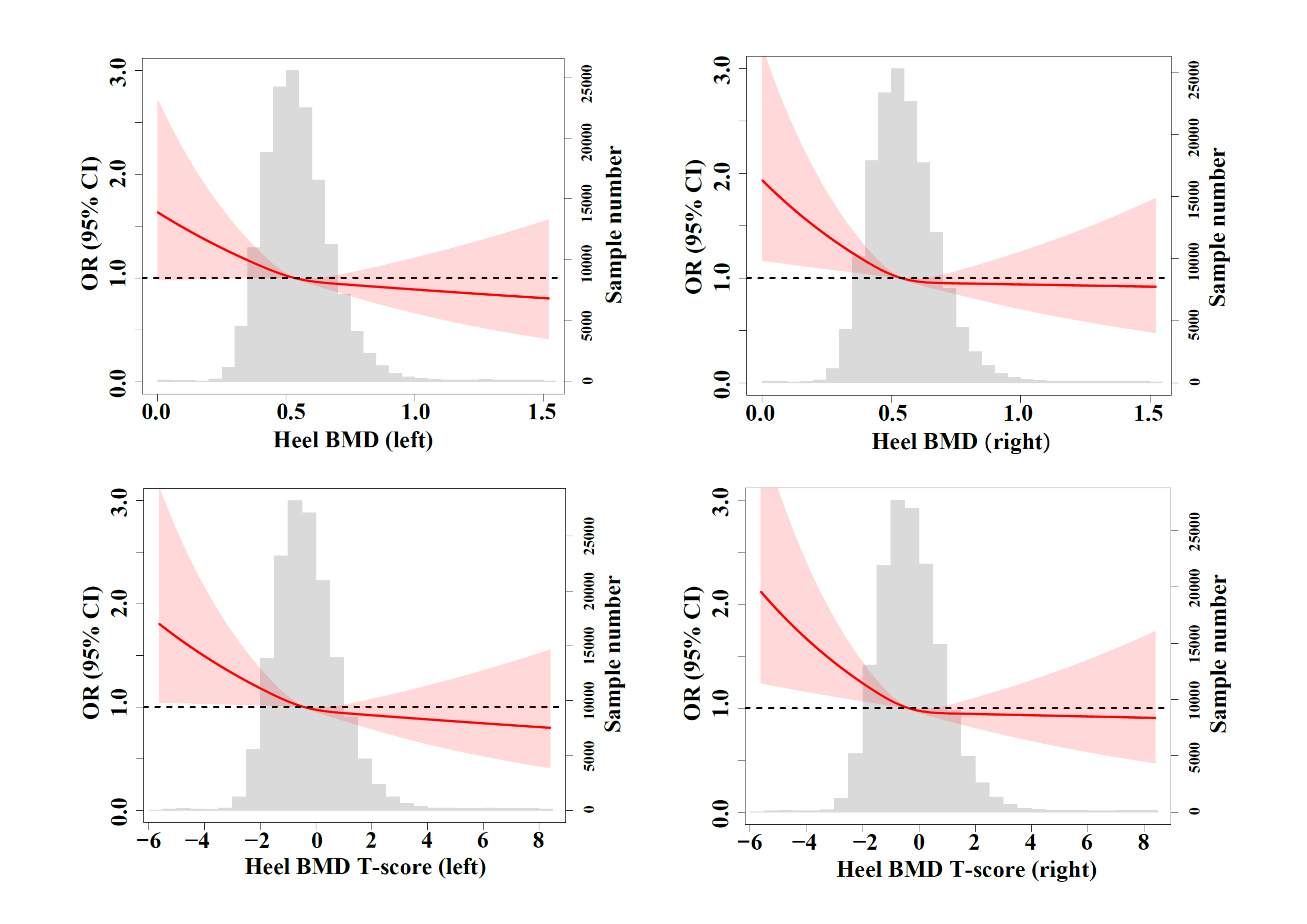


**Figure S1.** Exposure-response relationships between osteoporosis traits and sarcopenia. Exposure-response curves depicting odd ratios and 95% confidence intervals for sarcopenia risk against osteoporosis traits under restricted cubic spline regressions. Histograms display sample distributions of each trait. BMD: bone mineral density.

**
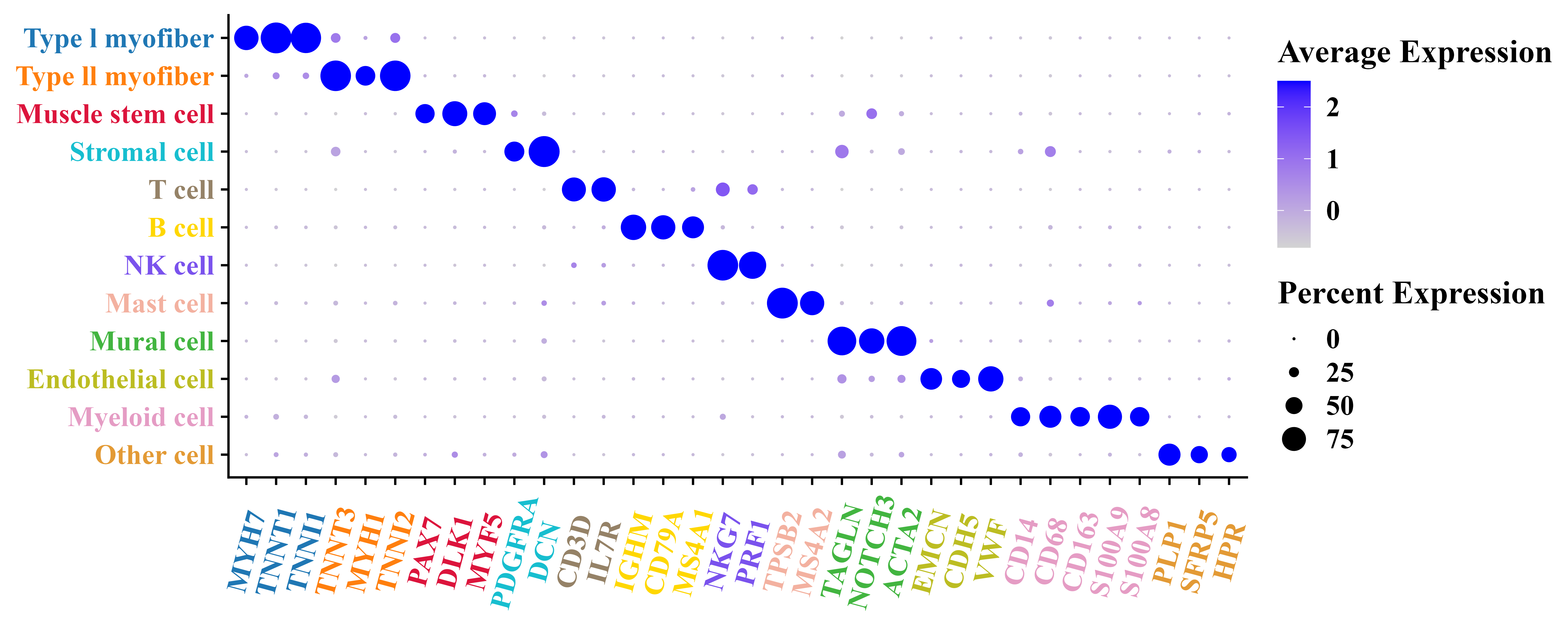
**

**Figure S2.** Marker gene expressions of different cell types in skeletal muscle. Average expression represents the average of standardized gene expression levels within each cell type. Percent expression denotes the percentage of cells expressing the given gene within each cell type.


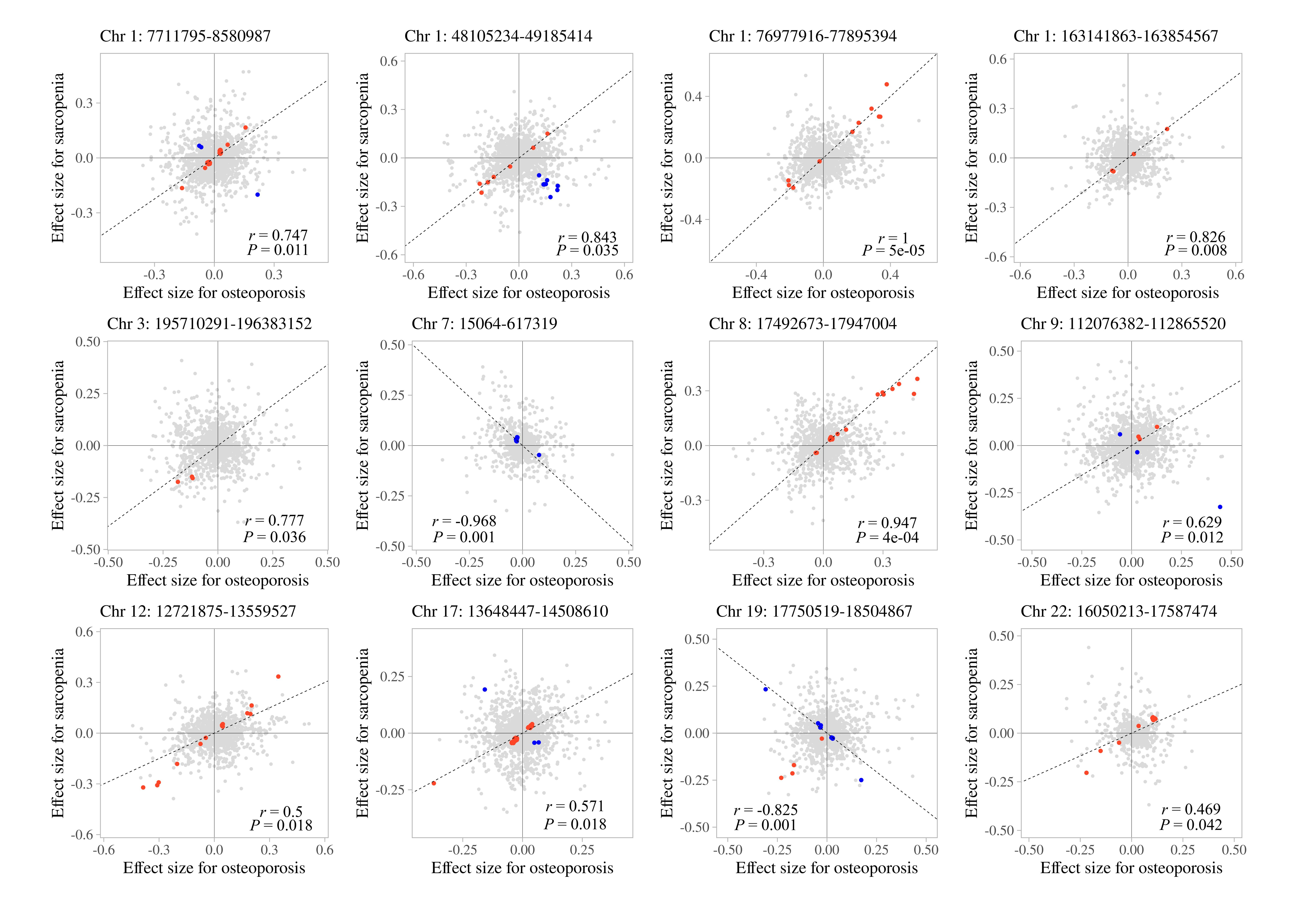


**Figure S3**. Scatter plots of SNP effect sizes for sarcopenia risk (x-axis) and osteoporosis risk (y-axis) in genomic regions with significant local genetic correlation. Red and bule dots denote SNPs significantly associated with both traits (*P* < 0.05) in consistent and opposite directions, respectively. Gray dots represent other SNPs in these regions. The slope of dashed line indicates the estimated local genetic correlation (*r*). *r* and *P* denote the local genetic correlation estimate and corresponding *P* value, respectively.


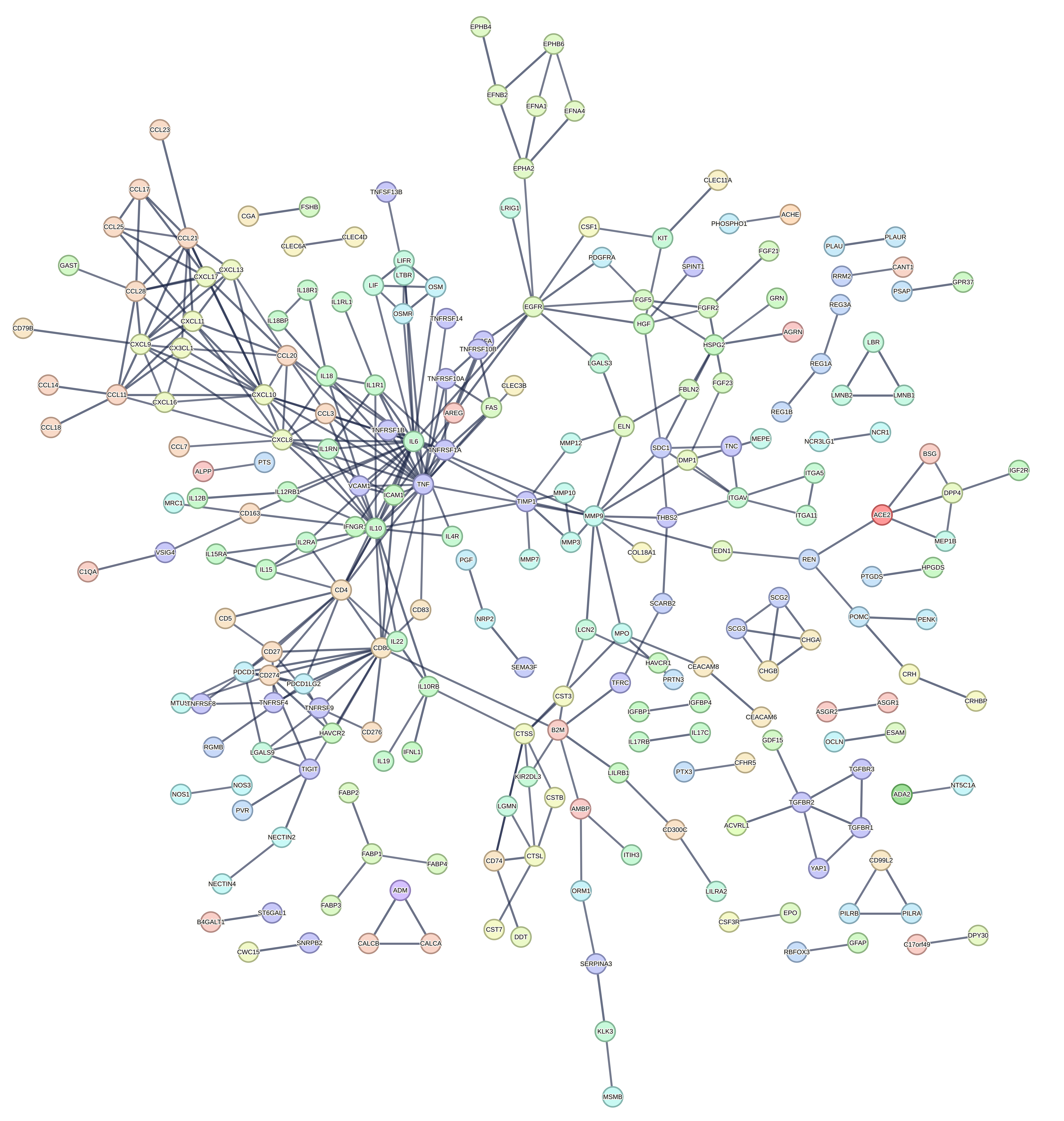


**Figure S4**. Protein-protein interaction (PPI) network for proteins associated with both osteoporosis and sarcopenia in consistent effect directions. The PPI network has a clustering coefficient of 0.318, containing 496 nodes and 353 edges (expected number of edges: 67, enrichment *P* value<1×10^-16^) .

**Figure S5**. Transcription factors enrichment profile of proteins associated with both osteoporosis risk and sarcopenia risk.

**

**

**Figure S6.** Associations of proteomic and metabolomic signatures for sarcopenia traits with osteoporosis risk. (A)-(B) Survival curves for osteoporosis onset stratified by (A) proteomic and (B) metabolomic scores for sarcopenia traits. (C) Forest plot of hazard ratios and 95% confidence intervals for the associations of proteomic and metabolomic scores of sarcopenia traits with osteoporosis risk.


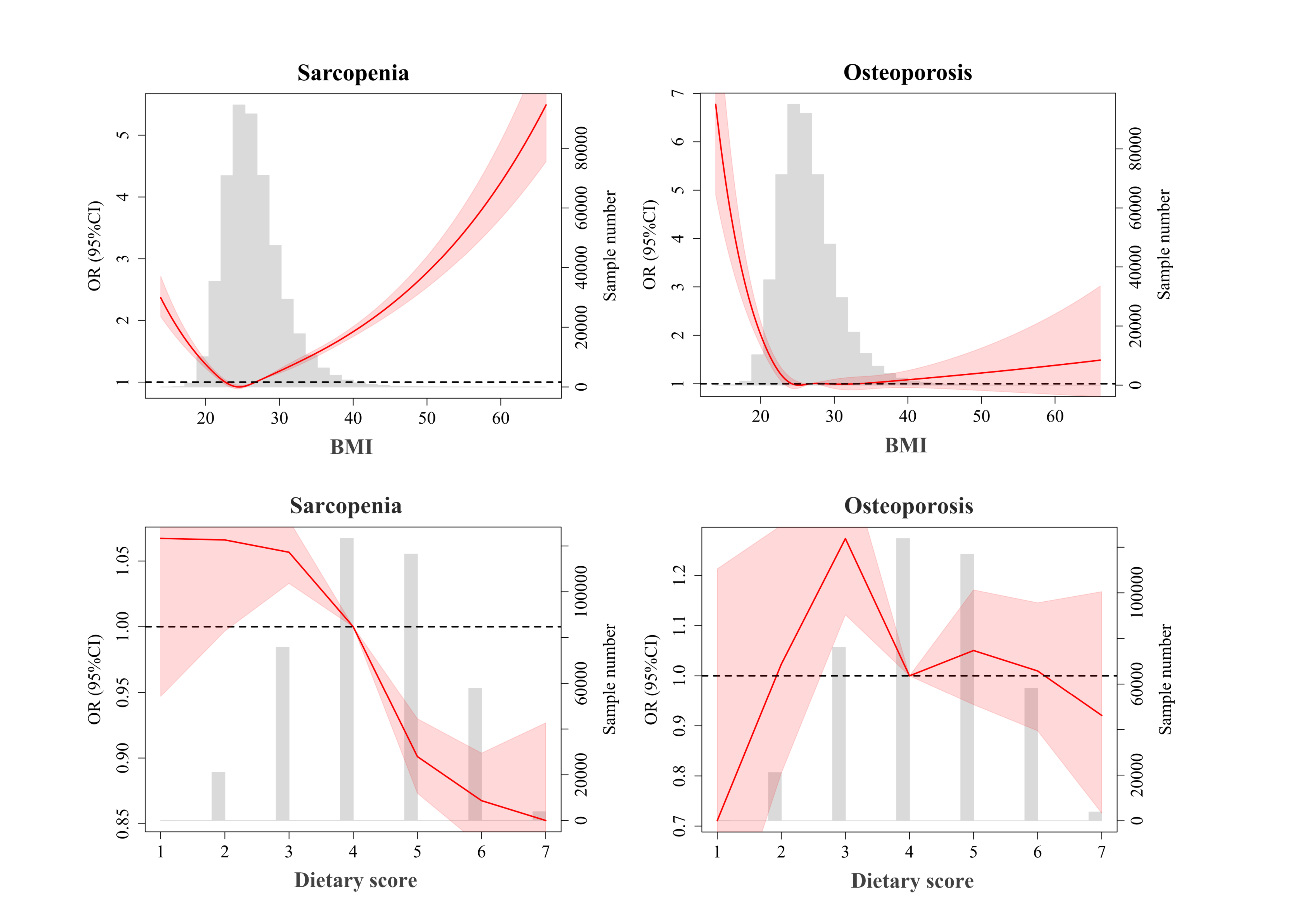
**Figure S7**. Exposure-response relationships between modifiable factors (body mass index and dietary score) and sarcopenia. Exposure-response curves depicting odds ratios and 95% confidence intervals for sarcopenia and osteoporosis risks against modifiable factors under restricted cubic spline regressions. Histograms display sample distributions of BMI and dietary score. BMI: body mass index.
